# Early life stress is associated with the default mode and fronto-limbic network connectivity among young adults

**DOI:** 10.3389/fnbeh.2022.958580

**Published:** 2022-09-23

**Authors:** Miro Ilomäki, Jallu Lindblom, Viljami Salmela, Marjo Flykt, Mervi Vänskä, Juha Salmi, Tuija Tolonen, Kimmo Alho, Raija-Leena Punamäki, Patrik Wikman

**Affiliations:** ^1^Department of Psychology and Logopedics, Faculty of Medicine, University of Helsinki, Helsinki, Finland; ^2^Faculty of Social Sciences/Psychology, Tampere University, Tampere, Finland; ^3^Department of Clinical Medicine, Faculty of Medicine, University of Turku, Turku, Finland; ^4^Department of Neuroscience and Biomedical Engineering, Aalto University, Helsinki, Finland; ^5^Advanced Magnetic Imaging Centre, Aalto NeuroImaging, Aalto University, Espoo, Finland

**Keywords:** early life stress (ELS), adverse childhood experience (ACE), functional connectivity, default mode network (DMN), fronto-limbic network

## Abstract

Exposure to early life stress (ELS) is associated with a variety of detrimental psychological and neurodevelopmental effects. Importantly, ELS has been associated with regional alterations and aberrant connectivity in the structure and functioning of brain regions involved in emotion processing and self-regulation, creating vulnerability to mental health problems. However, longitudinal research regarding the impact of ELS on functional connectivity between brain regions in the default mode network (DMN) and fronto-limbic network (FLN), both implicated in emotion-related processes, is relatively scarce. Neuroimaging research on ELS has mostly focused on single nodes or bi-nodal connectivity instead of functional networks. We examined how ELS is associated with connectivity patterns within the DMN and FLN during rest in early adulthood. The participants (*n* = 86; 47 females) in the current functional magnetic resonance imaging (fMRI) study were young adults (18–21 years old) whose families had participated in a longitudinal study since pregnancy. ELS was assessed both prospectively (parental reports of family relationship problems and mental health problems during pregnancy and infancy) and retrospectively (self-reported adverse childhood experiences). Inter-subject representational similarity analysis (IS-RSA) and multivariate distance matrix regression (MDMR) were used to analyze the association between ELS and the chosen networks. The IS-RSA results suggested that prospective ELS was associated with complex alterations within the DMN, and that retrospective ELS was associated with alterations in the FLN. MDMR results, in turn, suggested that that retrospective ELS was associated with DMN connectivity. Mean connectivity of the DMN was also associated with retrospective ELS. Analyses further showed that ELS-related alterations in the FLN were associated with increased connectivity between the prefrontal and limbic regions, and between different prefrontal regions. These results suggest that exposure to ELS in infancy might have long-lasting influences on functional brain connectivity that persist until early adulthood. Our results also speak for the importance of differentiating prospective and retrospective assessment methods to understand the specific neurodevelopmental effects of ELS.

## Introduction

Stressful and adverse experiences during pregnancy, infancy or childhood, conceptualized as early life stress (ELS), increase the risk for a wide range of somatic and mental health problems (Hughes et al., 2017). Children exposed to ELS are at a particularly heightened risk for self-regulation-related psychopathology (VanTieghem and Tottenham, 2018), characterized by altered stress responsivity (Dempster et al., 2021) and diminished ability to regulate one’s emotions (Burkholder et al., 2016).

Early development typically takes place in the context of a family, where infants and young children are entirely dependent on their primary caregivers. Sensitive parenting provides experiences of safety and successful co-regulation of arousal (Mueller and Tronick, 2019), and these in turn foster the development of adaptive physiological (e.g., hypothalamic-pituitary-adrenal axis) and emotional processes for the child (Rattaz et al., 2022). In contrast, if parenting is insensitive or unpredictable, the child may be exposed to overwhelming experiences of unregulated stress, with deleterious effects on development (Chen and Baram, 2016; DePasquale and Gunnar, 2020).

Importantly, parental mental health problems constitute a central risk for early parenting (Bernard et al., 2018). For example, parental depression is associated with high parenting stress (Leigh and Milgrom, 2008) and insensitive (e.g., harsh, coercive, or intrusive) parental behaviors (Lovejoy et al., 2000). Moreover, interparental problems tend to spill over to parent–child relationships, which can negatively impact the child’s well-being and development (Zhou et al., 2017). In addition, direct exposure to frequent interparental conflicts can be highly threatening for children and has negative consequences on cognitive and emotional development (Crockenberg et al., 2007; Pendry and Adam, 2013). Altogether, various stressors and problems within a child’s family environment can interfere with the development of adaptive emotion and stress regulation (Morris et al., 2007; Spangler and Zimmerman, 2014), both of which are important transdiagnostic processes underlying mental health (Hu et al., 2014; Fernandez et al., 2016). Altered development of self-regulation processes has been shown to mediate the effects of ELS on later mental health (Demir et al., 2020).

In human research, ELS is typically assessed as a cumulative sum of adverse and highly stressful experiences. The content of these stressors ranges from mental health problems in the family to blatant maltreatment (Finkelhor et al., 2015). Brain researchers acknowledge that a large portion of ELS studies is limited in using retrospective assessments (McLaughlin et al., 2019; Kraaijenvanger et al., 2020). Typically, in such retrospective studies, the participants report their recalled childhood experiences using self-report questionnaires. An alternative approach utilizes prospectively assessed ELS, based on, for example, official records or parental reports. Critically, retro- and prospectively assessed ELS shows poor convergence (Baldwin et al., 2019), especially regarding the more diffuse adverse experiences such as emotional neglect (Reuben et al., 2016). The issue is further complicated by adults lacking the ability to report events that occurred during their first 3 years of life, a phenomenon called childhood amnesia (Bauer, 2015). Consequently, retrospective ELS studies may have overemphasized events that occurred during later childhood. To better understand the potential causal role of ELS on brain development, it is important to evaluate whether the retro- and prospectively assessed ELS have different associations with functional brain connectivity. Thus, we will examine whether prospectively (i.e., parental reports of mental health, interparental, and parenting problems during the pregnancy and infancy) and retrospectively (i.e., young adults’ self-reports of adverse childhood experiences) assessed ELS are congruent in their influence on functional connectivity.

The importance of studying ELS in early development relates to the high developmental plasticity of the brain during this period. Neurodevelopmental research indicates that the first few years of life are crucial for the brain development underlying social and emotional capacities (Short and Baram, 2019). This early sensitive period is partly due to the rapid maturation of the prefrontal cortex, which is malleable to ELS (Hodel, 2018). Additionally, maternal experiences of distress (e.g., anxiety and depression) during pregnancy have been found to alter fetal brain development (Scheinost et al., 2020; Wu et al., 2020). A potential mechanism for such an effect is that fetal exposure to maternal stress hormones would increase neural plasticity, which in turn would make the child more vulnerable to postnatal environmental stressors such as interparental or parenting problems (O’Donnell and Meaney, 2017).

Research has identified ELS-related functional and structural neurodevelopmental alterations in and between various brain regions that relate to emotion processing, such as the prefrontal cortex, amygdala, and hippocampus (Cohen et al., 2006; Smith and Pollak, 2020; Chahal et al., 2021). While there is some evidence of ELS-related alterations in bi-nodal functional and structural brain connectivity (e.g., Philip et al., 2013; Cohodes et al., 2020), longitudinal studies focusing on alterations in network-level functional connectivity are still rare. Such an approach may better capture how multiple brain regions work together in the development of cognitive, psychophysiological, and emotional regulation. A network-level functional connectivity approach, compared to bi-nodal connectivity, is also more in line with the nature of the brain, as network-like structures are prevalent even at the level of neurons. In the current longitudinal study, we adopted a network-level approach to elucidate the impact of ELS on larger-scale functional connectivity. We focused on two self-regulation-related brain networks: the default mode network (DMN) and the fronto-limbic network (FLN).

The default mode network (DMN) is a functional brain network that is especially apparent during rest while no explicit tasks are being attended to (Raichle et al., 2001). Anatomically the DMN involves the medial prefrontal cortex (mPFC), posterior cingulate cortex (PCC), angular gyrus/inferior parietal cortex (IPC), dorsolateral prefrontal cortex (dlPFC), and middle temporal gyrus (MTG; Andrews-Hanna et al., 2014). As the PCC is the main input for the hippocampus, the DMN is often considered to extend to the medial temporal cortex (Alves et al., 2019). The DMN is related to a range of functions (Yeshurun et al., 2021), for example, thinking about oneself or others (Molnar-Szakacs and Uddin, 2013), remembering the past or planning the future (Xu et al., 2016; Huo et al., 2018), and regulating attention to external or internal stimuli (e.g., daydreaming or mind-wandering; Kucyi and Davis, 2014). A recent meta-analysis suggested that the DMN is the principal neural substrate of rumination, a form of dysfunctional involuntary emotion regulation (Nolen-Hoeksema et al., 2008; Zhou et al., 2020). Furthermore, alongside its many associations with psychopathology, decreased functional connectivity within the DMN has been associated with, for example, major depressive disorder (Whitfield-Gabrieli and Ford, 2012).

There is some evidence that ELS is associated with both the structure and function of the DMN. For example, Zeev-Wolf et al. (2019) found that ELS, as indicated by mothers’ mental health problems, insensitive parenting, and war-related traumatic events across early childhood, was associated with reduced DMN connectivity in preadolescent children. Studies have also found that post-traumatic stress disorder following childhood maltreatment is associated with reduced anterior-posterior integration within the DMN among adults (Daniels et al., 2011; DiGangi et al., 2016). Daniels et al. (2011) interpreted this reduced connectivity as reflecting an alteration in the maturation of the DMN. Correspondingly, Philip et al. (2013) found that in healthy adults, ELS was associated with decreased connectivity between PCC and mPFC. Importantly, delayed maturation of the DMN, especially decreased anterior-posterior integration, has been associated with psychopathology in children who have experienced ELS (Sato et al., 2015), which further elucidates the connection between ELS and later mental health problems.

The FLN, in turn, is a theoretically constructed network that encompasses the mPFC, anterior cingulate cortex (ACC), orbitofrontal cortex (OFC), nucleus accumbens, amygdala, and hippocampus. The treatment of the FLN as a distinct network is based on both anatomical connectivity between fronto-limbic regions, and on the evidence that the prefrontal cortex (PFC) is involved in the regulation of limbic activity in subcortical regions, such as the amygdala (Marek et al., 2013). The PFC has anatomical projections with numerous limbic regions such as the amygdala and hippocampus, which, in turn, are known to modulate the activity of the nucleus accumbens, one of the two nuclei comprising the ventral striatum implicated in reward-related processing (Arco and Mora, 2009). In addition to the amygdala and hippocampus, the nucleus accumbens has projections to frontal regions such as the ACC (Salgado and Kaplitt, 2015). The OFC, in turn, projects bidirectionally to the amygdala (Matyi and Spielberg, 2020), and the ACC has extensive projections to several brain regions implicated in emotion, autonomic function, reward processing, and memory, such as the OFC, amygdala, and hippocampus (Stevens et al., 2011). Finally, the hippocampus, which has been associated with especially autobiographical memory and spatial navigation (Squire, 1992; Buzsáki and Moser, 2013), appears to also engage in emotion processing (Santangelo et al., 2018; Zhu et al., 2019).

Altered connectivity between different frontal and limbic brain regions has been implicated in ELS and experiences of stress in general. Animal studies have demonstrated heterogenous stress-induced cellular and neural network alterations that influence how information is integrated between the PFC and limbic regions (Lee and Goto, 2011; Lee et al., 2011). The role of OFC activity and OFC-amygdala connectivity in punishment and fear-related learning has also been well documented in animals (Bukalo et al., 2015; Hsieh and Chang, 2020; Ma et al., 2020; Shih and Chang, 2021). In humans, structural OFC-amygdala connectivity has been shown to decrease following ELS (Goetschius et al., 2020). Functional connectivity between parts of the mPFC and with both amygdala and OFC correlate with successful emotion regulation (Banks et al., 2007). Peverill et al. (2019) showed that functional connectivity between the ventromedial prefrontal cortex and amygdala was weaker during an emotional task in adolescents who had prior experiences of physical, emotional, or sexual abuse. Further, Gee et al. (2013) have shown ELS-related decoupling between mPFC and amygdala, reflecting mPFC-amygdala connectivity seen in adults which may imply accelerated maturation of the circuitry. Decreased mPFC-amygdala functional connectivity has been well replicated in research on childhood trauma (Cisler, 2017), yet increases in fronto-limbic functional connectivity, for example, increased mPFC-amygdala (Philip et al., 2013) and functional connectivity have also been implicated in some ELS research (e.g., Philip et al., 2013), suggesting that inspections of bi-nodal connections might not capture the complexities underlying ELS related alterations in brain development. Finally, in their review, Cohodes et al. (2020) report a variety of structural and functional alterations to connectivity between frontal and limbic regions, especially when caregivers were involved in the ELS. Neurodevelopmental research into the influence of ELS in humans has often focused on narrower, bi-nodal fronto-limbic connectivity (e.g., mPFC-amygdala connectivity). Even though such bi-nodal connectivity has been associated with, for example, emotion regulation (Marek et al., 2013; Sicorello and Schmahl, 2021), the relation between ELS and fronto-limbic functional connectivity remains relatively elusive. The main focus of the current study on network-level functional connectivity attempts to determine this potentially complex relation by examining network-level patterns that analyses on bi-nodal connectivity might not be able to capture.

In the current study, our general aim was to examine the relationship between ELS and the resting-state activity within DMN and FLN in early adulthood. The reason for focusing on these networks stems from their relevancy in processing emotions, self-regulation, and psychopathology. The research data is from a Finnish 20-year longitudinal study called Miracles of Development (MIDE), in which the families have been followed from the mother’s pregnancy to the child’s early adulthood. ELS was measured prospectively during pregnancy and infancy (based on parental reports), and retrospectively in late adolescence (based on self-reports). Functional magnetic resonance imaging (fMRI) data were measured from the now-adult participants at the age of 18–21 years.

Our main hypothesis was that higher ELS is associated with altered connectivity within the DMN and FLN. We tested this (1) by studying the association between ELS and mean connectivity within the DMN and FLN. Because several studies (e.g., Banks et al., 2007; Goetschius et al., 2020) have implicated that ELS modulates connectivity between the amygdala and OFC, mPFC, we specifically tested whether prospective and retrospective ELS reflects the connectivity of these regions in the current dataset. It is important to note that the mean connectivity level does not exhaustively index nuances underlying the development of brain networks. Therefore, (2) we ran a further analysis applying inter-subject representational similarity analysis (IS-RSA) to model the associations of ELS with the DMN and FLN. This application of the original RSA allowed us to consider the multivariate nature of the fMRI data, that is, to test whether ELS is associated with complex connectivity patterns in these two networks. To better understand the patterns of network connectivity in IS-RSA, (3) we examined the individual pairwise connections between all nodes within the networks that were most strongly associated with ELS (either positively or negatively). Finally, (4) we conducted the analyses separately for the parental reports of ELS (during pregnancy and child’s infancy) and the self-reported ELS (in late adolescence) to scrutinize the role of prospective and retrospective assessments.

## Materials and methods

### Participants and procedure

The current study is part of a larger Finnish longitudinal project called MIDE. The project has followed 953 families since pregnancy. In approximately half of the families, the parents had successful assisted reproductive treatment (ART; 51%, *n* = 484) and in half, the child was naturally conceived (NC; 49%, *n* = 469). In the current study, resting-state fMRI data were collected from 92 young adults at the age of 18–21 years (*M* = 19.06, *SD* = 0.77; 55% female). The inclusion criteria for fMRI were being right-handed, native Finnish speaker with normal hearing, normal or corrected vision, and no current psychiatric or neurological illnesses. The young adults had provided self-report data at the age of 17–19 years (*M* = 18.23, *SD* = 0.34), and their parents provided self-report data during the child’s pregnancy (the 2nd trimester), and at the child’s age of 2 and 12 months. During pregnancy, the inclusion criteria for the parents were being Finnish speaking, and for the NC group, not having an infertility history, and mother’s age >25 years to correspond with the higher age of ART mothers. A more detailed description of the larger study sample is available elsewhere (e.g., Vänskä et al., 2011; Flykt et al., 2021).

The participants for the fMRI study were selected using a disproportionate stratified sampling (see Parsons, 2017). This was to ensure that the whole range of participants was well represented in the study sample. The sampling was based on the prospective ELS risk, composed of 20 variables indicating family relationship problems and mental health problems during pregnancy and child’s infancy (see section “Prospectively assessed early life stress”). Using this risk index, the larger study sample was divided into four equally sized strata (*z* < −0.42 for low-risk; −0.42 ≤ *z* < 0.24 for moderate-low-risk; 0.24 ≤ *z* < 0.90 for moderate-high-risk; 0.90 ≤ *z* for high-risk). We planned to sample 24 participants from each of the four strata (i.e., the total *n* = 96), balancing for child’s sex (50% girls, 50% boys) and parents’ infertility history (50% ART, 50% naturally conceived). We considered eligible only the cases which had a maximum of 8 (of a total of 20 variables) missing values in the prospective risk index. We achieved to sample *n* = 92 participants, with some deviations from our plan (e.g., some cells ran out and were replaced by nearby cells). Nonetheless, the sample successfully represented all the strata, χ^2^(3) = 0.35, *p* = 0.951, and was well-balanced for child’s sex, χ^2^(3) = 1.55, *p* = 0.671, and parents’ fertility history, χ^2^(3) = 0.21, *p* = 0.976, within the stratum.

Of the initial sample of 92 young adults, six were excluded from further analysis due to excessive head motion (>0.2 mm mean framewise displacement) during the fMRI procedure, resulting in a final sample of 86 young adults to be used in the analyses. The fMRI experiment, as well as the previous stages of the study, were approved by the Ethics Committee of the Hospital District of Helsinki and Uusimaa, Finland.

### Questionnaires

#### Prospectively assessed early life stress

Prospective ELS was assessed during pregnancy and at the child’s age of 2- and 12 months using questionnaires filled by both mothers and fathers. *Family mental health problems* were assessed using Beck’s Depression Inventory (BDI-13; for mothers: α’s = 0.75–0.84, for fathers: α’s = 0.80–0.83) (Beck et al., 1961) and General Health Questionnaire (GHQ-36; for mothers: α’s = 0.91–0.94, for fathers: α’s = 0.92–0.94) (Goldberg and Hillier, 1979) at all three time points. BDI and GHQ cover depression symptoms, and the GHQ also covers anxiety, insomnia, and social dysfunction. *Family relationship problems* were assessed using Dyadic Adjustment Scale (DAS; for mothers: α’s = 0.92–0.93, for fathers: α’s = 0.91–0.91) (Spanier, 1976) and Parenting Stress Index (PSI-36; for mothers: α’s = 0.90–0.90, for fathers: α’s = 0.91–0.91) (Abidin, 1997) at the child’s age of 2 and 12 months. DAS covers problems in the interparental relationship, such as conflicts and low affection, and PSI covers problems in the parent–child relationships, such as parenting distress and relational dysfunction. Complete prospective ELS data (i.e., 20 variables) were available for 84% (*n* = 77) of the participants, whereas 9% (*n* = 8) had eight missing variables and 7% (*n* = 7) had one to four missing variables. Missing variables were handled using Expectation-Maximization (EM) imputation using information from the larger study sample (see Participants and Procedure). To indicate the total ELS, a cumulative risk score (see Ettekal et al., 2019) was formed based on the 20 variables. For that, the scores of each questionnaire (e.g., BDI) were averaged over time, and parents, standardized, and averaged to form the total score (*M* = 0.00, *SD* = 0.86). The variables showed moderately high covariation (α’s = 0.88).

#### Retrospectively assessed early life stress

Retrospective ELS was operationalized using self-report questionnaire items derived from the revised inventory of Adverse Childhood Experiences (Finkelhor et al., 2015). The self-report data was collected approximately 1 year prior to fMRI measurement when participants were 17–19 years old (*M* = 18.23, *SD* = 0.34). Some additional items were also created based on research literature to capture common ELS events. Two items (with a three-point Likert scale, 0 = never, 1 = sometimes, 2 = often) were used to assess the following domains: Emotional abuse (e.g., “Did a parent or other adult in the household often or very often swear at, insult, or put you down?”); Physical abuse (e.g., “Did a parent or other adult in the household often or very often push, grab, shove, or slap you?”); Emotional neglect (e.g., “Did you often or very often feel that no one in your family loved you or thought you were important or special?”); Parent treated violently (e.g., “Was your parent often or very often pushed, grabbed, slapped, or had something thrown at her/him?”); Interparental psychological violence (e.g., “Have you seen your parent being threatened by violence at home”; Ellonen et al., 2008). We also used one-item questions (with a binary response scale, 0 = no, 2 = yes) to assess the following domains: Family alcohol and drug problem (“Did you live with anyone who was a problem drinker or alcoholic, or who used street drugs?”); Peer victimization (“Have you been bullied in school?”); Parents’ divorce (“Did your parents separate/divorce?”); Family mental illness (“Was a household member mentally ill?”); Death of a close person (“Have you ever lost anyone close to you by death?”); Family somatic illness (“Has some of your family members had a serious illness during your life?”); and Other serious adversities (“Have you experienced other adversities, such as accidents, victimization, or natural catastrophes?”). To form a total sum, scores for the two-item domains were averaged, and subsequently, the sum of the 12 domains was computed (*M* = 4.45, *SD* = 2.60; range = 0–11.50).

#### Covariates

Family socioeconomic status (SES) and mother’s age [in years; measured in 2018 when young adults provided self-report data (see Section “Retrospectively assessed early life stress”)], and family fertility history (0 = ART, 1 = NC) and participant sex (0 = girl, 1 = boy) were used as covariates. SES was indexed as mother’s education using 5-level grading (0 = university level education, 4 = no vocational education).

### Functional magnetic resonance imaging data

#### MRI data acquisition

All data were acquired using a 3T MAGNETOM Skyra whole-body scanner (Siemens Healthcare, Erlangen, Germany) with a 20-channel head coil at the Advanced Magnetic Imaging (AMI) Centre, Aalto NeuroImaging, Aalto University School of Science, Espoo, Finland. Before collecting the resting-state data, participants completed other tasks during fMRI. The first task was a go/no-go task that required the participants to respond to different facial expressions (neutral, happy, or angry; adapted from Hare et al., 2008). The second task involved photographs of faces cropped to show eyes only (adapted from Moor et al., 2012). In two conditions, the participants had to pick a word that either describes (1) the thoughts or feelings of the person in the photograph, or (2) the gender and age of the person in the photograph. Next, an anatomical scan [MPRAGE; high-resolution 3D T1 anatomical images (voxel matrix 256 × 256, in-plane resolution 1 mm × 1 mm × 1 mm)] was obtained. This anatomical image was used in the current study. Next, participants completed a social media task in which they posted opinions to a bogus Facebook group created by the experimenters and received peer feedback on those opinions (see Wikman et al., 2021). Finally, we acquired resting-state data (participants were asked to lie still with eyes open) using echo-planar imaging (EPI) with an imaging area covering the whole brain comprising 43 contiguous oblique slices (TR 2,500 ms, TE 32 ms, flip angle 75°, voxel matrix 64 × 64, field of view 20 cm, slice thickness 3.0 mm, in-plane resolution 3.1 mm × 3.1 mm × 3.0 mm). Participants were reimbursed 15 €/hour (2–3 h) for their time.

#### Functional magnetic resonance imaging pre-processing

Preprocessing was carried out using fMRIprep pipelines, one resulting in co-registered preprocessed data on the fsaverage surface, and the other in each individual’s T1w space. Next, we’ll describe the processes carried out by fMRIprep in detail. First, head-motion parameters were estimated with respect to a sample fMRI volume, henceforth be referred to as the BOLD reference. During this step, the full fMRI dataset was motion corrected in respect to the BOLD reference using mcflirt (FSL 5.0.9, Jenkinson et al., 2002; transformation matrices, and six corresponding rotation and translation parameters). Next, fMRI data were slice-time corrected using 3dTshift from AFNI 20160207 (Cox and Hyde, 1997, RRID:SCR_005927). The BOLD reference was then co-registered to the T1w image generated from each individual’s anatomical scan (see Section “MRI data acquisition”) using bbregister (FreeSurfer) which implements boundary-based registration (Greve and Fischl, 2009). Co-registration was configured with six degrees of freedom. To generate confound time series, used to denoise the fMRI data (see below), the fMRI time series were resampled into standard MNI152NLin6Asym space (note: this data was only used to generate confound time series used to denoise the resting-state data). ICA-AROMA (Pruim et al., 2015) was performed on the preprocessed fMRI data in MNI space after removal of non-steady state volumes and spatial smoothing with an isotropic, Gaussian kernel of 6 mm^3^ FWHM (full-width half-maximum). Several confounding time series were calculated: framewise displacement (FD) and three region-wise global signals. FD is calculated for each functional run, using its implementation in Nipype (following the definitions by Power et al., 2014). The three global signals are extracted within the CSF, the WM, and the whole-brain masks.

Gridded (volumetric) resamplings were performed using antsApplyTransforms (ANTs), configured with Lanczos interpolation to minimize the smoothing effects of other kernels (Lanczos, 1964). Brain surfaces were reconstructed from the participants’ T1 images using recon-all (FreeSurfer 6.0.1, RRID:SCR_001847, Dale et al., 1999). The preprocessed fMRI time series in T1w space were resampled onto the fsaverage surface. FMRI time series in fsaverage space were denoised in the following way: first, they were smoothed with Gaussian kernel of 6 mm^2^ FWHM. Then, ‘non-aggressive’ denoising using ICA-AROMA was performed using the components generated in the previous preprocessing step. Additionally, global signals from WM, CSF, and the whole-brain mask were regressed out and the time series were high-pass filtered using a discrete cosine filter with 128 s cut-off. FMRI time-series in T1w space were denoised in a similar way, but without any spatial smoothing. This resulted in denoised fMRI data in fsaverage space used to generate cortical ROI time series (see Section “Construction of functional networks”), and denoised fMRI data in T1w-space used to generate subcortical ROI time series (see Section “Construction of functional networks”).

### Construction of functional networks

DMN was constructed using parcels from the HCP’s multi-modal parcellation, version 1.0 (HCP_MMP1.0; Glasser et al., 2016) for the cortical regions of interest (ROIs). FSL’s FIRST segmentation (Patenaude et al., 2011) was used for the subcortical ROIs in the DMN. The five ROIs were constructed separately for the left and right hemispheres, resulting in 10 separate cortical ROIs. For subcortical ROIs, the hippocampus was selected from the FIRST segmentation, resulting in a total of 12 ROIs for the DMN. A list of parcels for the ROIs in DMN can be found in Table 1. FLN was constructed by using parcels from the HCP_MMP1.0 atlas for the cortical ROIs. FSL’s FIRST segmentation (Patenaude et al., 2011) was used for the subcortical ROIs in the FLN. For subcortical ROIs, we selected the hippocampus, amygdala, and accumbens-area from the FIRST segmentation, resulting in a total of 12 ROIs for the FLN. A list of parcels used for the ROIs in FLN can be found in Table 2. Functional connectivity was obtained by first calculating the average time series for each ROI using the preprocessed data (fsaverage resampled data for the cortical regions and T1 resampled data for the subcortical regions). For both networks, Pearson’s correlations of time series between each parcel were calculated with Nilearn toolbox (Abraham et al., 2014), resulting in a 12 × 12 correlation matrix for both the DMN and FLN.

**TABLE 1:** List of parcels from the HCP_MMP1.0 atlas and the subcortical ROIs from FSL’s FIRST segmentation combined to form the six ROIs for the default mode network.

PCC	mPFC	IPC	MTG	DLPFC	HC
7m	s32	PGi	TE1a	8Av	17
31pd	a24	PGs	TE1p	8C	53
31pv	p32	PFm	TE2a		
d23ab	10r		TE1m		
v23ab	10d				
31a	9m				
23d					
POS1					

PCC, posterior cingulate cortex; mPFC, medial prefrontal cortex; IPC, inferior parietal cortex; MTG, middle temporal gyrus; DLPFC, dorsolateral prefrontal cortex; HC, hippocampus.

**TABLE 2:** List of parcels from the HCP_MMP1.0 atlas and the subcortical ROIs from FSL’s FIRST segmentation combined to form the six ROIs for the fronto-limbic network.

OFC	mPFC	ACC	AMG	NAcc	HC
11l	s32	p24	18	26	17
13l	a24	a24pr	54	58	53
47m	p32	p24pr			
47s	10r				
10v	10d				
OFC	9m				
10pp					

OFC, orbitofrontal cortex; mPFC, medial prefrontal cortex; ACC, anterior cingulate cortex; AMG, amygdala; NAcc, nucleus accumbens; HC, hippocampus.

### Analyses of functional connectivity

First, to gauge potentially simple associations between ELS and functional connectivity, a univariate Spearman correlation was calculated between the independent risk variables and the averaged DMN and FLN connectivity vectors. We also conducted simple linear regression analyses for the OFC- and mPFC-amygdala connectivity with prospective ELS and retrospective ELS as independent variables separately. All of the regression models had the covariates: mother’s age and SES, participant sex, and ART history. In total, 16 simple regression analyses were conducted and the results were corrected using false discovery rate (FDR).

Second, representational similarity analysis (RSA; Kriegeskorte et al., 2008) was used to capture fine differences within the DMN and FLN connectivity structures between participants based on their exposure to stressful events in early life. RSA is a computational technique that is used to reveal higher-order representational spaces by utilizing pairwise comparisons of different stimuli. In the current study, we utilized IS-RSA (inter-subject representational similarity analysis; Finn et al., 2020) in pairwise comparisons between participants’ DMN and FLN connectivity structures instead. The representational dissimilarity matrices (RDM) used in the analysis, which typically characterize the information carried by a given representation in the brain, characterize here the difference in the connectivity structure of the given network (DMN or FLN) between participants. The RDMs used in the current study, therefore, display the similarities/differences between all participants’ connectivity profiles. RDMs were created for the connectivity of the DMN and FLN, for the prospective and retrospective ELS scores, and for relevant background variables (mother’s age and SES, participant sex, and ART history, see Supplementary Figure 1). We also made additional model RDMs for the different subscales comprising the prospective ELS (Supplementary Figures 2, 3). The connectivity RDMs were created by simply subtracting the correlations of participant profiles from one. The ELS and other model RDMs were created by calculating the absolute pairwise differences in the scores of all participants.

Third, using partial correlation to control for the effect of the background variables, the RDMs were correlated to see whether participants who were similar based on their ELS scores were also similar in their DMN and FLN connectivity profiles. Since correlation is very sensitive to outliers, outliers that were two standard deviations below or above the mean of the participants were discarded from the analyses (DMN mean connectivity: five participants; DMN IS-RSA: five participants; FLN mean connectivity: three participants; FLN IS-RSA: five participants). To better understand the influence of the prospective ELS index, additional correlations were calculated between the network connectivity RDMs and new model RDMs created by using only individual components of the prospective ELS index [family (RDAS, PSI) vs. mental health related (BDI, GHQ); BDI, GHQ, RDAS and PSI individually; ELS at T1, T2, and T3 individually; mother’s and father’s scores individually]. To better understand differences in connectivity profiles, we created circulographs displaying the most different pairwise connections in the DMN and FLN by comparing those with the most ELS to those with the least.

Fourth, we performed multivariate distance matrix regression (MDMR; Anderson, 2001; McArdle and Anderson, 2001) using the statistical software R (R Core Team, 2022) to further test the association between ELS and the brain networks of interest. Both of the models (one for each brain network) had the covariates: mother’s age and SES, participant sex, and ART history. Similarly as the IS-RSA, the MDMR tests the multivariate association between the dissimilarity/distance matrix of the participants and the independent variables, but MDMR uses different statistic than IS-RSA, and thus it complements the IS-RSA analysis.

## Results

### Univariate connectivity analyses

**FIGURE 1:**
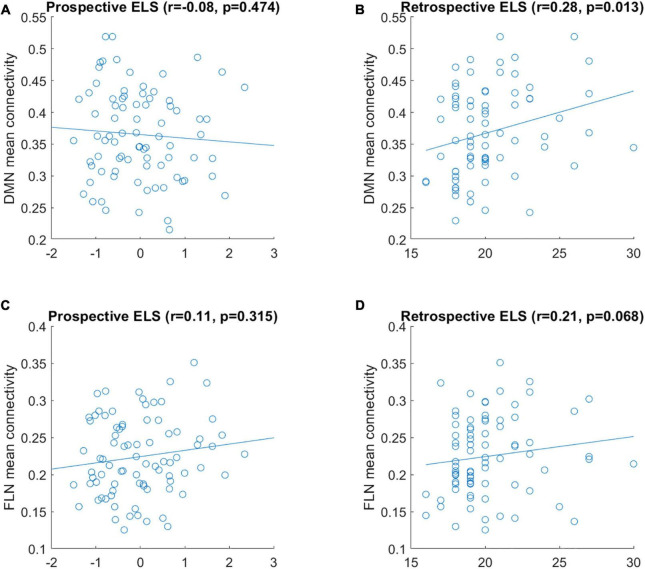
Correlation between mean connectivity of DMN **(A,B)** and FLN **(C,D)** and prospective ELS **(A,C)** and retrospective ELS **(B,D)** variables. *y*-axis represents the mean correlation strength between the nodes of the network and *x*-axis represents the scores obtained from the ELS measure.

Simple univariate correlations between the ELS scores and the mean connectivity of DMN and FLN networks are displayed in Figure 1. There were no significant correlations between prospective ELS scores and the mean connectivity of the DMN (*r* = −0.08, *p* = 0.474; Figure 1A) or FLN (*r* = 0.11, *p* = 0.315; Figure 1C). However, there was a significant correlation between retrospective ELS and the mean connectivity of the DMN (*r* = 0.28, *p* = 0.013; Figure 1B), but not of the FLN (*r* = 0.21, *p* = 0.068; Figure 1D). After controlling for background variables (mother’s SES and age, participant sex and ART history), the DMN × Retrospective ELS correlation remained significant (*r* = 0.25, *p* = 0.043). Steiger’s test for dependent correlations showed that the correlation between mean DMN connectivity and prospective ELS, and the correlation between mean DMN connectivity and retrospective ELS were significantly different (*z* = −2.393, *p* = 0.017). In FLN, the correlation between mean connectivity and retrospective ELS and prospective ELS did not differ (*z* = −0.626, *p* = 0.53).

Additionally, we conducted simple linear regression analyses for the OFC- and mPFC-amygdala connectivity separately with prospective ELS and retrospective ELS as independent variables. Significant effects of retrospective risk were found for the right OFC–right amygdala [*t*(86) = 3.00, *p* = 0.004, *d* = 0.32], left OFC–right amygdala [*t*(86) = 2.52, *p* = 0.014, *d* = 0.27], and left OFC–right amygdala [*t*(86) = 2.48, *p* = 0.015, *d* = 0.26] connectivity. Note, however, that none of the results remained significant after FDR correction (*p* = 0.06; *p* = 0.08, and *p* = 0.08, respectively). For prospective risk, no significant effects were found (all *t* < 1.80, *p* > 0.07). None of the regression analyses involving the mPFC–amygdala connections yielded significant results (all *t* < 1.8, *p* > 0.08).

### Network structure

**FIGURE 2:**
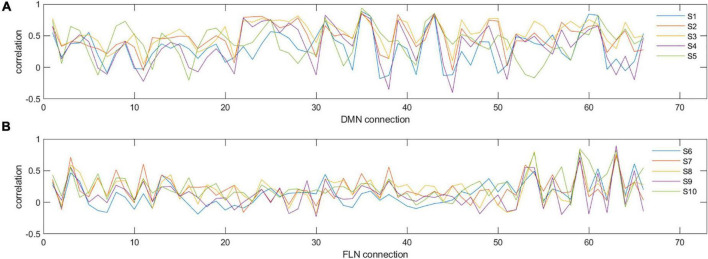
Examples of individual connectivity ‘profiles’ in DMN **(A)** and FLN **(B)** for five selected participants (S1–S5 for DMN and S6–S10 for FLN). *y*-axis represents the correlation strength and *x*-axis represents each of the different pairwise correlations of all the nodes in that network. As can be seen, certain nodes show very consistent correlations across participants, while others show variability. For example, for the DMN, connectivity measure numbers 31, 35, and 36 are consistent across the five participants (reflecting similarity), whereas connectivity measure numbers 30, 32, 33, and 34 show high variability (reflecting dissimilarity).

Due to the network mean connectivity being a rather coarse measure, we next investigated how prospective and retrospective ELS scores are associated with the whole network structure. To this end, we utilized IS-RSA and RDMs. In IS-RSA, each participant has a network connectivity profile comprising a vector for all pairwise connections within the network. Thus, for both the DMN and FLN, a single vector consisted of 66 values (pairwise connections of 12 regions). This is exemplified in Figures 2A,B showing connectivity profiles of 5 participants for the DMN and FLN, respectively.

**FIGURE 3:**
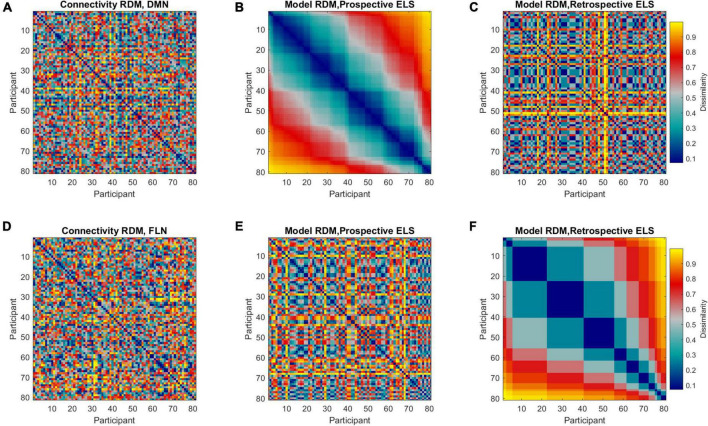
Representational dissimilarity matrices (RDMs) representing dissimilarities between all pairwise comparisons across all participants (81 × 81, *n* = 86, 5 outliers removed). Colors indicate the extent of dissimilarity between participants, where dark blue represents maximum similarity and yellow represents maximum dissimilarity. Representational dissimilarity matrices are displayed for DMN **(A)** and FLN **(D)** connectivity profiles, prospective ELS **(B,E)**, and retrospective ELS **(C,F)**. Note that matrices **(A–C)** are sorted according to prospective ELS, and matrices **(D–F)** are sorted according to retrospective ELS.

Representational dissimilarity matrices were calculated separately for both the DMN and FLN, the prospective and retrospective ELS variables, and background variables (i.e., mother’s age and SES, ART history, participant sex). The connectivity and ELS RDMs are displayed in Figure 3. The RDMs were calculated by correlating the connectivity profiles across all participants and subtracting the correlation from one, resulting in 81 × 81 RDMs. The RDMs represent how similar/dissimilar each participant is compared to every other participant on that variable.

**FIGURE 4:**
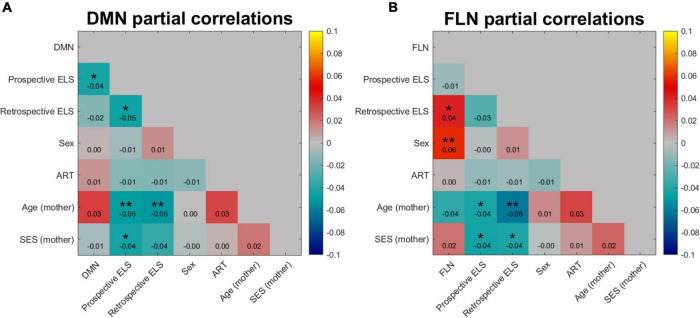
Partial correlation of DMN **(A)** and FLN **(B)** network profile dissimilarities with dissimilarity matrices of prospective and retrospective ELS, with participant sex, assisted reproduction treatment (ART) history, mother’s age, and mother’s socio-economic status (SES) partialled out. Colors indicate the strength and direction of correlation between the dissimilarity matrices of variables, where blue represents negative correlations and red and yellow represent positive correlations. *p < 0.05, **p < 0.01.

The model RDMs (reflecting dissimilarities in ELS scores) were correlated with connectivity RDMs (reflecting dissimilarities in connectivity structure of the DMN and FLN), revealing 3rd level correlations. Model RDMs were also calculated for all relevant background variables (see Supplementary Figure 1) and the sub-scales of the prospective ELS variable (Supplementary Figures 2, 3). The partial correlations between connectivity and model RDMs are displayed in Figure 4. The DMN RDM and the prospective ELS RDM had a significant correlation (*r* = −0.04, *p* = 0.02), as did the FLN RDM and the retrospective ELS RDM (*r* = 0.04, *p* = 0.03) and FLN RDM and sex of the participant (*r* = 0.06, *p* = 0.002). The non-partial correlations between network connectivity and Prospective ELS (DMN *r* = −0.04; FLN *r* = −0.002) and between network connectivity and Retrospective ELS (DMN *r* = −0.02; FLN *r* = 0.04) did not differ (Steiger’s test, DMN *z* = −0.5, *p* = 0.62; FLN *z* = −1.79, *p* = 0.07).

Since the DMN × Prospective ELS and FLN × Retrospective ELS associations are 3rd level correlations, the interpretation of the effect is not straightforward. These results do not imply that changes in the ELS variables increase or decrease total connectivity within DMN or FLN. Instead, these results indicate that the connectivity structure of the networks, as a whole, changes as a function of ELS.

**FIGURE 5:**
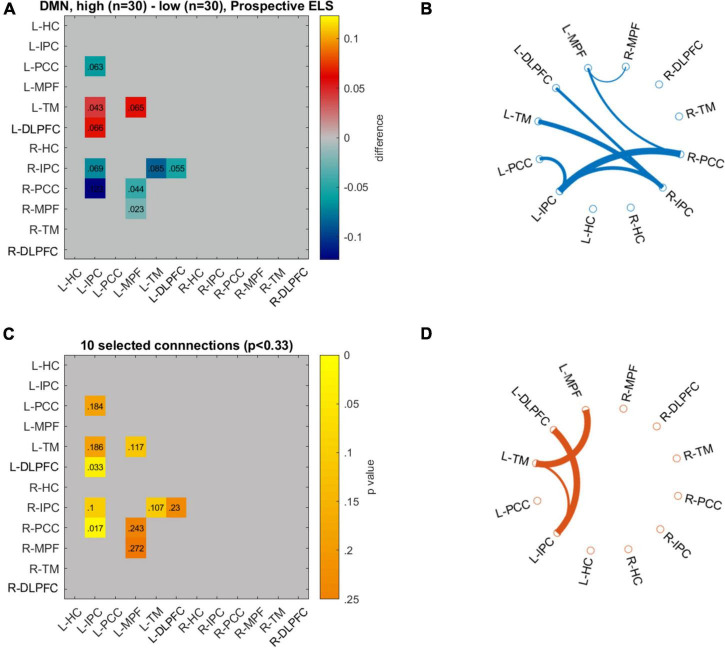
Differences in network connectivity between participants with highest (*n* = 30) and lowest (*n* = 30) prospective ELS values are plotted as mean difference **(A)** and 10 connections with the lowest *p*-values **(C)**. Circulographs of the negative (**B**; high prospective ELS → reduced connectivity) and positive (**D**; high prospective ELS → increased connectivity) differences.

**FIGURE 6:**
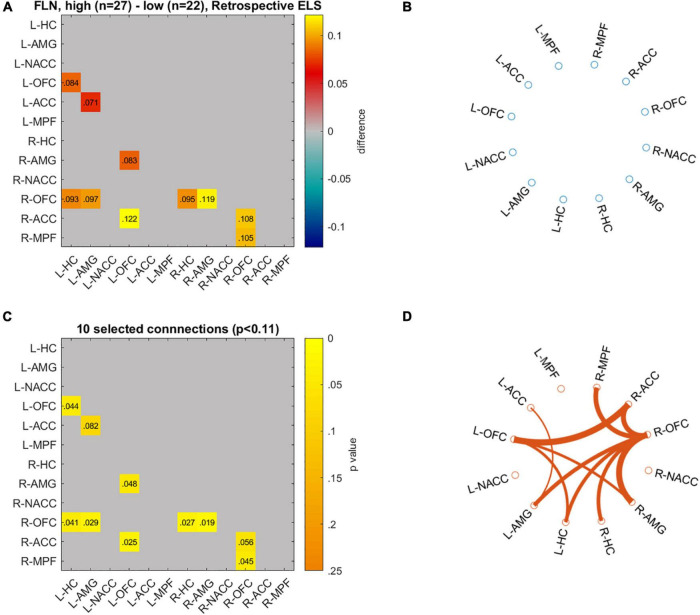
Differences in network connectivity between participants with highest (*n* = 27) and lowest (*n* = 22) retrospective ELS values are plotted as mean difference **(A)** and *p*-values for these connections **(C)**. Circulographs of the negative (**B**; high retrospective ELS → reduced connectivity) and positive (**D**; high retrospective ELS → increased connectivity) differences.

We visualized the data with circulographs (Figures 5, 6) by comparing the connectivity between those with the lowest and highest scores on the two ELS measures. For the DMN network, where there was a significant association between the DMN RDMs and the prospective ELS RDMs, the circulographs revealed that, on average, some connections were slightly stronger and some were weaker for participants with low prospective risk vs. high prospective risk, but the differences were not significant. For the FLN, in turn, where there was a significant association between the retrospective ELS RDMs and the FLN RDMs, the circulographs revealed that this was related to the fact that for participants with high retrospective ELS, several connections in the network showed stronger connectivity than for participants with low retrospective ELS. Note that the circulographs were used for visualization purposes, since none of the displayed *p*-values in the circulographs remained significant after FDR correction.

In the MDMR analyses (Tables 3, 4), retrospective ELS was significantly associated with DMN connectivity (*pseudo r*^2^ = 0.03; *p* = 0.014). Significant effects were not found for the association between FLN and prospective ELS (*p* = 0.53) or retrospective ELS (*p* = 0.17) in the MDMR. Thus, while IS-RSA showed an association between DMN and prospective ELS as well as FLN and retrospective ELS, the MDMR showed an association between DMN and retrospective ELS (similar to correlation analysis of the mean connectivity).

**TABLE 3:** Multivariate distance matrix regression (MDMR) analysis results for the default mode network (DMN), with prospective ELS and retrospective ELS as independent variables, and participant sex, ART, mother’s age and mother’s SES as covariates.

DMN	Statistic	*df*	Pseudo *R*^2^	*p*-value
(Omnibus)	0.1428	6	0.12495	0.008**
Prospective ELS	0.0069	1	0.00602	0.952
Retrospective ELS	0.0352	1	0.03083	0.014*
Participant sex	0.0240	1	0.02102	0.098
ART	0.0192	1	0.01684	0.216
Mother’s age	0.0428	1	0.03748	<0.002***
Mother’s SES	0.0136	1	0.01190	0.452

*p < 0.05, **p < 0.01, ***p < 0.001.

**TABLE 4:** Multivariate distance matrix regression (MDMR) analysis results for the fronto-limbic network (FLN), with prospective ELS and retrospective ELS as independent variables, and participant sex, ART, mother’s age and mother’s SES as covariates.

FLN	Statistic	*df*	Pseudo *R*^2^	*p*-value
(Omnibus)	0.1110	6	0.0999	0.088
Prospective ELS	0.0126	1	0.0114	0.526
Retrospective ELS	0.0200	1	0.0180	0.174
Participant sex	0.0272	1	0.0245	0.050*
ART	0.0174	1	0.0157	0.260
Mother’s age	0.0122	1	0.0110	0.542
Mother’s SES	0.0172	1	0.0155	0.230

*p < 0.05.

### Additional analyses

To clarify the source of how the prospective ELS index modulated DMN, we compared the network RDMs to model RDMs of the sub-variable used in constructing the prospective ELS index. Of all the sub-variable RDMs, parents’ mental health (comprising all BDI and GHQ scores from both parents), parents’ BDI alone, ELS at T1, ELS at T2, and fathers’ scores RDMs were all associated with the DMN RDM, while BDI, and GHQ RDMs were associated with FLN RDM (Supplementary Figures 4–8).

## Discussion

In the current study, we analyzed how ELS was associated with functional brain connectivity within both the DMN and FLN. The novelty in our study relates to examining the possible functional alterations in distributed brain network patterns and to evaluating whether retrospectively (i.e., adolescent self-reported childhood adversities) and prospectively (i.e., pre- and postnatal family relationship problems and parental mental health problems) assessed ELS are associated with different connectivity patterns. Prospectively, but not retrospectively, assessed ELS was positively correlated with mean DMN connectivity. Yet, neither ELS index was associated with mean FLN connectivity. *Ad hoc* analyses on bi-nodal connectivity between the amygdala and OFC, mPFC suggested an association between higher retrospectively assessed ELS and increased OFC-amygdala functional connectivity, yet these results were non-significant after FDR correction. The IS-RSA revealed an association between the prospective ELS and DMN connectivity structure, and between retrospective ELS and FLN connectivity structure. The MDMR revealed an association between retrospective ELS and DMN connectivity structure. Inspection of pairwise connections with circulographs showed that for the FLN, higher retrospective ELS was associated with stronger connectivities, which likely contributed to the IS-RSA result. However, inspection of the pairwise connections with circulographs did not explain the association between prospective ELS and DMN. Altogether, the results support our hypothesis that ELS is associated with alterations in these functional networks. The results further indicate that the alterations in connectivity following ELS are complex and that retrospectively and prospectively assessed ELS are differently associated with functional brain connectivity in the DMN and FLN.

In line with our hypotheses, we found that ELS was associated with changes in functional connectivity of the DMN at the level of both mean connectivity (retrospective), IS-RSA (prospective), and MDMR (retrospective). Many previous studies on ELS and DMN functional connectivity have reported decreased connectivity (Daniels et al., 2011; Philip et al., 2013; DiGangi et al., 2016; Zeev-Wolf et al., 2019). Thus, it was surprising that our mean level analyses showed an association between retrospective ELS and increased rather than decreased DMN connectivity. The reason for this discrepancy is not clear, but it may relate to the timing and content of the measured stressors. As suggested by our additional analyses on the ELS subdomains, the association between prospective ELS and DMN connectivity structure was mostly driven by the very early stressors (i.e., during pregnancy T1, and at the child’s age of 2 months T2), parental depression (i.e., BDI), and the father’s (rather than mother’s) responses (see Supplementary Figures 4–8). The discrepancy might also be due to unique vulnerability and resilience processes present in different study populations. Indeed, most of the previous research concerns individuals exposed to war-related events who also tend to suffer from post-traumatic symptoms (Daniels et al., 2011; DiGangi et al., 2016; Zeev-Wolf et al., 2019). It is not unlikely that exposure to such extreme events has different neural consequences compared to exposure to chronic family-related stressors. More on par with our study, Philip et al. (2013) focused on western adults. However, they excluded participants with ongoing mental health problems, potentially emphasizing the presence of more resilient individuals in the sample. Indeed, our results converge with the broader research on mental health showing that increased DMN connectivity is associated with both psychopathology and rumination (Whitfield-Gabrieli and Ford, 2012; Zhou et al., 2020). However, some recent research has also identified decreased functional connectivity within the DMN in patients with recurrent depression (Yan et al., 2019). This incongruency suggests that interactions between behavior and network-level functional connectivity are complex. This proposition is supported by our IS-RSA results, as the association between prospective ELS and the DMN connectivity structure appears complex as well.

Even though the early parent- and family-related stressors were associated with alterations in the functional structure of the DMN, such alterations need to be associated with behavioral outcomes to evaluate whether the changes are detrimental, beneficial, or neutral. Due to the complexity of the alterations, it will be important in future studies to consider that ELS may lead to multiple patterns of altered DMN. For example, the different patterns could reflect the fundamentally different ways children respond and cope with the overwhelming stress in their family (Davies et al., 2016), or more general individual differences in temperamental resiliency against the stress (Belsky, 2013). These patterns are worth exploring further in future research due to the relevancy of the DMN in processes relating to psychopathology. Future research must also focus on the precise operationalization of stressors to elucidate their impact on the development of the DMN and its relation to psychopathology at different age period, for example, infancy, middle childhood, and adolescence. Because sensitive parenting relates to the development of adaptive physiological stress-regulation systems (e.g., hypothalamic-pituitary-adrenal axis; Rattaz et al., 2022), future research could also benefit from incorporating biomarkers of stress into their analyses of the influence of ELS on brain development.

Retrospectively assessed ELS was associated with FLN but not DMN connectivity structure in IS-RSA. Hence, those who report similar ELS retrospectively are also more similar in terms of their FLN connectivity profile. The MDMR analysis, however, showed an association between retrospective ELS and DMN. Inspection of pairwise linear connections illustrated that higher ELS was associated with widespread increases in connectivity between many of the nodes in the FLN. Interestingly, every single strengthened pairwise connection in the limbic regions occurred with PFC regions, while some connections between different PFC regions were also strengthened. This strengthening of fronto-limbic connectivity is contradictory to findings from studies on more extreme ELS, where PFC-amygdala connectivity has been found to decrease as a result of extreme childhood adversities (Cisler, 2017; Goetschius et al., 2020). Assuming that fronto-limbic functional connectivity is associated with regulatory functions, this finding also contradicts some studies that have associated deficiencies in emotion regulation with decreased fronto-limbic functional connectivity (Sicorello and Schmahl, 2021). Yet, our results are in line with other findings that report increased connectivity between PFC regions (e.g., mPFC) and limbic regions (e.g., amygdala). In particular, Philip et al. (2013) also reported increased mPFC-amygdala connectivity in their sample of 22 medication-free healthy adults. Similar to the study by Philip et al. (2013), our sample reflected a relatively healthy population of young adults. This observation supports the interpretation that resilience in addition to latent vulnerabilities needs to be considered when inspecting the association between ELS and brain development.

As our results demonstrate, different operationalizations of ELS can have very different associations with specific outcome variables such as functional connectivity within a brain network. Our prospective ELS variable captured problems in parental mental health and family relations across the pregnancy and infancy, and thus reflected chronic stressors in the early family climate. Our retrospective ELS variable, in turn, captured young adults’ recollections of adverse events across childhood and adolescence. To some extent, the retrospective ELS index consisted of items that reflect more severe and prominent ELS than the prospective index. At the same time, however, measuring ELS retrospectively in adulthood is prone to subjectivity biases such as infantile amnesia and motivational biases. Indeed, depression, for example, is characterized by negative cognitive biases related to self and others (Weeks et al., 2017; Orchard and Reynolds, 2018), which is likely to result in more negative evaluations of past childhood experiences by depressed people. Thus, to some extent, the association between retrospective ELS and FLN connectivity may reflect a depressive perception of own past, rather than neurodevelopmental alterations caused by actual ELS. This issue is further complicated, however, by that ELS can genuinely increase depressive thinking, leading to exaggerated perceptions of ELS. While further studies are required to scrutinize this issue, our results demonstrate that the prospective and retrospective ELS have highly different associations with functional connectivity patterns.

While not the primary focus of the current study, it is important to note that half of our participants were conceived with assisted reproductive treatment (ART). Some studies have reported a negative impact of infertility history and ART on parental mental health and early family relations (Fisher et al., 2005; Monti et al., 2015), and ART-related early embryological and epigenetic processes may impact fetal and child brain development (Boutet et al., 2021; Håberg et al., 2022). However, our results yielded no ART-related alterations in neither FLN nor DMN functional connectivity. This is an important contribution to ART literature and indicates no discernable differences related to ART and family infertility history.

On the broader level, our pattern of results relating to the association between retrospectively measured ELS and network connectivity aligns with the view that a child’s subjective interpretation of stressors might be more important for brain development than the actual stressor itself (Smith and Pollak, 2020). From preventive and promotive perspective, it is important to note that such subjective interpretations can be influenced by specific parental socialization practices. For example, it has been shown that children can learn conditioned fear vicariously by simply observing their parents react to the conditioned stimulus (Marin et al., 2020). Some infants might be more at risk of the influences of stressors in early life due to their parents’ reactivity to stressors. This pattern of reactivity in the child’s parents might pass on to the child, resulting in sensitivity to these specific external influences and creating vulnerability to later mental health issues as a result. Future studies on the impact of ELS on development could benefit from considering how specific parental practices in the family may modulate children’s ways to interpret and perceive stressors, and to potentially reveal ways to increase children’s resiliency against stressors.

The strengths of the current study include a relatively large study sample, stratified sampling, both prospective and retrospective assessments of ELS, and the inspection of network-level functional connectivity. Yet, our study also has several limitations. First, our study population may reflect a healthier population compared to many other high-risk studies on ELS, some of which have also included clinical groups. Incorporating these groups into future studies on the impact of ELS might help capture underlying resilience or vulnerability factors that influence the association between ELS exposure and functional brain connectivity. Second, while we did adopt a novel approach by inspecting network-level functional connectivity alterations with IS-RSA, our results using the MDMR analysis also showed an association between ELS and network connectivity but provided somewhat different results. While this incongruency serves as an interesting result in itself, as it highlights how different statistical choices can lead to varying, there was also agreement between them in that an association between ELS and DMN connectivity was found with both analyses. It is also noteworthy that our application of IS-RSA for studying functional connectivity is relatively novel and some uncertainty about its true applicability remains open to debate. Reliable methods need to be incorporated into future neurodevelopmental studies to elucidate the potentially complex association between ELS and network-level patterns of change in functional connectivity. Third, developmental mediating or moderating factors during the time period after infancy were not considered. These factors might constitute protective or harmful influences that might alter how ELS influences brain development from infancy into early adulthood.

## Conclusion

Our study showed that ELS is associated with alterations in DMN and FLN functional connectivity and that the association was dependent on the way ELS was operationalized and statistically tested. Our results support the notion that ELS is a heterogenous and dynamic phenomenon that must be meticulously operationalized to capture its complex influence on psychological and brain development. Incongruent results between different operationalizations of ELS suggest a need for more specific language about and operationalization of ELS, as well as more nuanced network-level statistical approaches, in future research.

## Data availability statement

The datasets presented in this article are not readily available because participant privacy and ethical permissions related to this data do not allow public sharing of the data. Requests to access the datasets should be directed to R-LP, raija-leena.punamaki-gitai@tuni.fi.

## Ethics statement

The studies involving human participants were reviewed and approved by Ethics Committee of the Hospital District of Helsinki and Uusimaa, Finland. Written informed consent to participate in this study was provided by the participants’ legal guardian/next of kin.

## Author contributions

R-LP, JL, MV, and MF designed the project and collected the longitudinal data. PW collected the fMRI data. TT processed the fMRI data. MI, JL, VS, PW, and JS designed the analyses, analyzed the data, and interpreted the data. MI and JL wrote the manuscript with support from all other authors. All authors approved the submitted version.

## References

[B1] Abidin R. R. (1997). “Parenting stress index: A measure of the parent–child system,” in *Evaluating Stress: A Book of Resources*, eds Zalaquett C. P. Wood R. J. (Scarecrow Education).

[B2] Abraham A. Fabian P. Michael E. Gervais P. Mueller A. Kossaifi J. (2014). Machine learning for neuroimaging with scikit-learn. *Front. Neuroinform.* 8:14. 10.3389/fninf.2014.00014 24600388 PMC3930868

[B3] Alves P. N. Foulon C. Karolis V. Bzdok D. Margulies D. S. Volle E. (2019). An improved neuroanatomical model of the default-mode network reconciles previous neuroimaging and neuropathological findings. *Commun. Biol.* 2:370. 10.1038/s42003-019-0611-3 31633061 PMC6787009

[B4] Anderson M. J. (2001). A new method for non-parametric multivariate analysis of variance. *Austral Ecol.* 26 32–46. 10.1111/j.1442-9993.2001.01070.pp.x

[B5] Andrews-Hanna J. Smallwood J. Spreng N. (2014). The default network and self-generated thought: component processes, dynamic control, and clinical relevance. *Ann. N.Y Acad. Sci.* 1316 29–52. 10.1111/nyas.12360 24502540 PMC4039623

[B6] Arco A. D. Mora F. (2009). Neurotransmitters and prefrontal cortex–limbic system interactions: Implications for plasticity and psychiatric disorders. *J. Neural Trans.* 116 941–952. 10.1007/s00702-009-0243-8 19475335

[B7] Baldwin J. R. Reuben A. Newbury J. B. Danese A. (2019). Agreement between prospective and retrospective measures of childhood maltreatment: A systematic review and meta-analysis. *JAMA Psychiatry* 76 584–593. 10.1001/jamapsychiatry.2019.0097 30892562 PMC6551848

[B8] Banks S. J. Eddy K. T. Angstadt M. Nathan P. J. Phan K. L. (2007). Amygdala–frontal connectivity during emotion regulation. *Soc. Cogn. Affect. Neurosci.* 2 303–312. 10.1093/scan/nsm029 18985136 PMC2566753

[B9] Bauer P. J. (2015). A complementary processes account of the development of childhood amnesia and a personal past. *Psychol. Rev.* 122:204. 10.1037/a0038939 25844874

[B10] Beck A. T. Ward C. H. Mendelson M. Mock J. Erbaugh J. (1961). An inventory for measuring depression. *Arch. General Psychiatry* 4 561–571. 10.1001/archpsyc.1961.01710120031004 13688369

[B11] Belsky J. (2013). Differential susceptibility to environmental influences. *Int. J. Child Care Educ. Policy* 7 15–31. 10.1007/2288-6729-7-2-15

[B12] Bernard K. Nissim G. Vaccaro S. Harris J. L. Lindhiem O. (2018). Association between maternal depression and maternal sensitivity from birth to 12 months: a meta-analysis. *Attachment Hum. Dev.* 20 578–599. 10.1080/14616734.2018.1430839 29374991

[B13] Boutet E. Ahumada-Droguett P. Crovetto F. Cívico M. S. Manau D. (2021). P–766 neurodevelopment in fetuses conceived by assisted reproductive technologies following fresh and frozen embryo transfer. *Hum. Reproduct.* 36:765. 10.1093/humrep/deab130.765

[B14] Bukalo O. Pinard C. R. Silverstein S. Brehm C. Hartley N. D. Whittle N. (2015). Prefrontal inputs to the amygdala instruct fear extinction memory formation. *Sci. Adv.* 1:e1500251. 10.1126/sciadv.1500251 26504902 PMC4618669

[B15] Burkholder A. R. Koss K. J. Hostinar C. E. Johnson A. E. Gunnar M. R. (2016). Early life stress: Effects on the regulation of anxiety expression in children and adolescents. *Soc. Dev.* 25 777–793. 10.1111/sode.12170 28584408 PMC5454775

[B16] Buzsáki G. Moser E. (2013). Memory, navigation and theta rhythm in the hippocampal-entorhinal system. *Nat. Neurosci.* 16 130–138. 10.1038/nn.3304 23354386 PMC4079500

[B17] Chahal R. Kirshenbaum J. S. Ho T. C. Mastrovito D. Gotlib H. I (2021). Greater age-related changes in white matter morphometry following early life stress: Associations with internalizing problems in adolescence. *Dev. Cogn. Neurosci.* 47:100899. 10.1016/j.dcn.2020.100899 33340790 PMC7750321

[B18] Chen Y. Baram T. (2016). Toward understanding how early-life stress reprograms cognitive and emotional brain networks. *Neuropsychopharmacology* 41 197–206. 10.1038/npp.2015.181 26105143 PMC4677123

[B19] Cisler J. M. (2017). Childhood trauma and functional connectivity between amygdala and medial prefrontal cortex: A dynamic functional connectivity and large-scale network perspective. *Front. Syst. Neurosci.* 11:29. 10.3389/fnsys.2017.00029 28553208 PMC5425605

[B20] Cohen R. A. Grieve S. Hoth K. F. Paul R. H. Sweet L. Tate D. (2006). Early life stress and morphometry of the adult anterior cingulate cortex and caudate nuclei. *Biol. Psychiatry* 59 975–982. 10.1016/j.biopsych.2005.12.016 16616722

[B21] Cohodes E. M. Kitt E. R. Baskin-Sommers A. Gee D. G. (2020). Influences of early-life stress on frontolimbic circuitry: Harnessing a dimensional approach to elucidate the effects of heterogeneity in stress exposure. *Dev. Psychol.* 63 157–172. 10.1002/dev.21969 32227350

[B22] Cox R. W. Hyde J. S. (1997). Software tools for analysis and visualization of fMRI data. *NMR Biomed.* 10 171–178. 10.1002/(SICI)1099-1492(199706/08)10:4/5<171::AID-NBM453<3.0.CO;2-L 9430344

[B23] Crockenberg S. C. Leerkes E. M. Lekka S. K. (2007). Pathways from marital aggression to infant emotion regulation: the development of withdrawal in infancy. *Infant Behav. Dev.* 30 97–113. 10.1016/j.infbeh.2006.11.009 17292783

[B24] Dale A. M. Fischl B. Sereno M. I. (1999). Cortical surface-based analysis: I. segmentation and surface reconstruction. *NeuroImage* 9 179–194. 10.1006/nimg.1998.0395 9931268

[B25] Daniels J. K. Frewen P. McKinnon M. C. Lanius R. A. (2011). Default mode alterations in posttraumatic stress disorder related to early-life trauma: A developmental perspective. *J. Psychiatry Neurosci.* 36 56–59. 10.1503/jpn.100050 20964955 PMC3004976

[B26] Davies P. T. Martin M. J. Sturge-Apple M. L. (2016). Emotional security theory and developmental psychopathology. *Dev. Psychopathol.* 66:106. 10.1002/9781119125556.devpsy106 PMC3918896 24342849

[B27] Demir Z. Böge K. Fan Y. (2020). The role of emotion regulation as a mediator between early life stress and posttraumatic stress disorder, depression and anxiety in Syrian refugees. *Trans. Psychiatry* 10:371. 10.1038/s41398-020-01062-3 33139699 PMC7606478

[B28] Dempster K. O’Leary D. MacNeil A. Hodges G. Wade T. (2021). Linking the hemodynamic consequences of adverse childhood experiences to an altered HPA axis and acute stress response. *Brain Behav. Immun.* 93 254–263. 10.1016/j.bbi.2020.12.018 33358983

[B29] DePasquale C. E. Gunnar M. R. (2020). Parental sensitivity and nurturance. *Future Child.* 30 53–70.

[B30] DiGangi J. A. Tadayyon A. Fitzgerald D. A. Rabinak C. A. Kennedy A. Klumpp H. (2016). Reduced default mode network connectivity following combat trauma. *Neurosci. Lett.* 615 37–43. 10.1016/j.neulet.2016.01.010 26797653 PMC4810776

[B31] Ellonen N. Kääriäinen J. Salmi V. Sariola H. (2008). Lasten ja nuorten väkivaltakokemukset [Children’s and adolescents’ experiences of violence]. *Oikeuspoliittinen Tutkimuslaitos [Finland’s Natl. Res. Instit. Legal Policy]* 2008:152529.

[B32] Ettekal I. Eiden R. D. Nickerson A. B. Schuetze P. (2019). Comparing alternative methods of measuring cumulative risk based on multiple risk indicators: Are there differential effects on children’s externalizing problems? *PLoS One* 14:e0219134. 10.1371/journal.pone.0219134 31269048 PMC6609027

[B33] Fernandez K. C. Jazaieri H. Gross J. J. (2016). Emotion regulation: A transdiagnostic perspective on a nnew RDoC domain. *Cogn. Ther. Res.* 40 426–440. 10.1007/s10608-016-9772-2 27524846 PMC4979607

[B34] Finkelhor D. Shattuck A. Turner H. Hamby S. (2015). A revised inventory of adverse childhood experiences. *Child Abuse Neglect* 48 13–21. 10.1016/j.chiabu.2015.07.011 26259971

[B35] Finn E. S. Glerean E. Khojandi A. Y. Nielson D. Molfese P. J. Handweker D. A. (2020). Idiosynchrony: From shared responses to individual differences during naturalistic neuroimaging. *NeuroImage* 215:116828. 10.1016/j.neuroimage.2020.116828 32276065 PMC7298885

[B36] Fisher J. R. W. Hammarberg K. Baker H. W. G. (2005). Assisted conception is a risk factor for postnatal mood disturbance and early parenting difficulties. *Fertility Sterility* 84 426–430. 10.1016/j.fertnstert.2005.02.016 16084885

[B37] Flykt M. Vänskä M. Punamäki R.-L. Heikkilä L. Tiitinen A. Poikkeus P. (2021). Adolescent attachment profiles are associated with mental health and risk-taking behavior. *Front. Psychol.* 12:761864. 10.3389/fpsyg.2021.761864 34925164 PMC8674949

[B38] Gee D. G. Gabard-Durnam L. J. Flannery J. Goff B. Humphreys K. L. Telzer E. H. (2013). Early developmental emergence of human amygdala-prefrontal connectivity after maternal deprivation. *Proc. Natl. Acad. Sci. U.S.A.* 110 15638–15643. 10.1073/pnas.1307893110 24019460 PMC3785723

[B39] Glasser M. F. Coalson T. S. Robinson E. C. Hacker C. D. Harwell J. Yacoub E. (2016). A multi-modal parcellation of human cerebral cortex. *Nature* 536 171–178. 10.1038/nature18933 27437579 PMC4990127

[B40] Goetschius L. G. Hein T. C. Mitchell C. Lopez-Duran N. L. McLoyd V. C. Brooks-Gunn J. (2020). Childhood violence exposure and social deprivation predict adolescent amygdala-orbitofrontal cortex white matter connectivity. *Dev. Cogn. Neurosci.* 45:100849. 10.1016/j.dcn.2020.100849 32890959 PMC7481532

[B41] Goldberg D. P. Hillier V. F. (1979). A scaled version of the general health questionnaire. *Psychol. Med.* 9 139–145. 10.1017/S0033291700021644 424481

[B42] Greve D. N. Fischl B. (2009). Accurate and robust brain image alignment using boundary-based registration. *NeuroImage* 48 63–72. 10.1016/j.neuroimage.2009.06.060 19573611 PMC2733527

[B43] Håberg S. Page C. M. Lee Y. Nustad H. E. Magnus M. C. Haftorn K. L. (2022). DNA methylation in newborns conceived by assisted reproductive technology. *Nat. Commun.* 13:1896. 10.1038/s41467-022-29540-w 35393427 PMC8989983

[B44] Hare T. A. Tottenham N. Galvan A. Voss H. U. Glover G. H. Casey B. J. (2008). Biological substrates of emotional reactivity and regulation in adolescence during an emotional go-nogo task. *Biol. Psychiatry* 63 927–934. 10.1016/j.biopsych.2008.03.015 18452757 PMC2664095

[B45] Hodel A. S. (2018). Rapid infant prefrontal cortex development and sensitivity to early environmental experience. *Dev. Rev.* 48 113–144. 10.1016/j.dr.2018.02.003 30270962 PMC6157748

[B46] Hsieh H. Chang C. (2020). Activation of medial orbitofrontal cortex abolishes fear extinction and interferes with fear expression in rats. *Neurobiol. Learn. Memory* 165:107170. 10.1016/j.nlm.2020.107170 31978551

[B47] Hu T. Zhang D. Wang J. Mistry R. Ran G. Wang X. (2014). Relation between emotion regulation and mental health: A meta-analysis review. *Psychol. Rep.* 114 341–362. 10.2466/03.20.PR0.114k22w4 24897894

[B48] Hughes K. Bellis M. A. Hardcastle K. A. Sethi D. Butchart A. Mikton C. (2017). The effect of multiple adverse childhood experiences on health: A systematic review and meta-analysis. *Lancet Public Health* 2 e356–e366. 10.1016/S2468-2667(17)30118-4 29253477

[B49] Huo L. Li R. Wang P. Zheng Z. Li J. (2018). The default mode network supports episodic memory in cognitively unimpaired elderly individuals: Different contributions to immediate recall and delayed recall. *Front. Aging Neurosci.* 10:6. 10.3389/fnagi.2018.00006 29416508 PMC5787535

[B50] Jenkinson M. Bannister P. Brady M. Smith S. (2002). Improved optimization for the robust and accurate linear registration and motion correction of brain images. *NeuroImage* 17 825–841. 10.1006/nimg.2002.1132 12377157

[B51] Kraaijenvanger E. J. Pollok T. M. Monninger M. Kaiser A. Brandeis D. Banaschewski T. (2020). Impact of early life adversities on human brain functioning: A coordinate-based meta-analysis. *Neurosci. Biobehav. Rev.* 113 62–76. 10.1016/j.neubiorev.2020.03.008 32169412

[B52] Kriegeskorte N. Mur M. Bandettini P. (2008). Representational similarity analysis - connecting the branches of systems neuroscience. *Front. Syst. Neurosci.* 2:4. 10.3389/neuro.06.004.2008 19104670 PMC2605405

[B53] Kucyi A. Davis K. D. (2014). Dynamic functional connectivity of the default mode network tracks daydreaming. *NeuroImage* 100 471–480. 10.1016/j.neuroimage.2014.06.044 24973603

[B54] Lanczos C. (1964). Evaluation of noisy data. *J. Soc. Indust. Appl. Mathe. Seri. Numerical Anal.* 1 76–85. 10.1137/0701007

[B55] Lee Y.-A. Goto Y. (2011). Chronic stress modulation of prefrontal cortical NMDA receptor expression disrupts limbic structure–prefrontal cortex interaction. *Eur. J. Neurosci.* 34 426–436. 10.1111/j.1460-9568.2011.07750.x 21692885

[B56] Lee Y. A. Poirier P. Otani S. Goto Y. (2011). Dorsal-ventral distinction of chronic stress-induced electrophysiological alterations in the rat medial prefrontal cortex. *Neuroscience* 183 108–120. 10.1016/j.neuroscience.2011.03.039 21440605

[B57] Leigh B. Milgrom J. (2008). Risk factors for antenatal depression, postnatal depression and parenting stress. *BMC Psychiatry* 8:1–11. 10.1186/1471-244X-8-24 18412979 PMC2375874

[B58] Lovejoy M. C. Graczyk P. A. O’Hare E. Neuman G. (2000). Maternal depression and parenting behavior: A meta-analytic review. *Clin. Psychol. Rev.* 20 561–592. 10.1016/S0272-7358(98)00100-7 10860167

[B59] Ma C. Jean-Richard-dit-Bressel P. Roughley S. Vissel B. Balleine B. W. Killcross S. (2020). Medial orbitofrontal cortex regulates instrumental conditioned punishment, but not pavlovian conditioned fear. *Cerebral Cortex Commun.* 1:tgaa039. 10.1093/texcom/tgaa039 34296108 PMC8152850

[B60] Marek R. Strobel C. Bredy T. W. Sah P. (2013). The amygdala and medial prefrontal cortex: Partners in the fear circuit. *J. Physiol.* 591 2381–2391. 10.1113/jphysiol.2012.248575 23420655 PMC3678031

[B61] Marin M. F. Bilodeau-Houle A. Morand-Beaulieu S. Brouillard A. Herringa R. J. Milad M. R. (2020). Vicarious conditioned fear acquisition and extinction in child–parent dyads. *Sci. Rep.* 10:17130. 10.1038/s41598-020-74170-1 33051522 PMC7555483

[B62] Matyi M. A. Spielberg J. M. (2020). Differential spatial patterns of structural connectivity of amygdala nuclei with orbitofrontal cortex. *Hum. Brain Mapp.* 42 1391–1405. 10.1002/hbm.25300 33270320 PMC7927308

[B63] McArdle B. H. Anderson M. J. (2001). Fitting multivariate models to community data: A comment on distance-based redundancy analysis. *Ecology* 82 290–297. 10.1890/0012-96582001082[0290:FMMTCD]2.0.CO;2

[B64] McLaughlin K. A. Weissman D. Bitrán D. (2019). Childhood adversity and neural development: A systematic review. *Ann. Rev. Dev. Psychol.* 1 277–312. 10.1146/annurev-devpsych-121318-084950 32455344 PMC7243625

[B65] Molnar-Szakacs I. Uddin L. Q. (2013). Self-processing and the default mode network: Interactions with the mirror neuron system. *Front. Hum. Neurosci.* 7:571. 10.3389/fnhum.2013.00571 24062671 PMC3769892

[B66] Monti F. Agostini F. Paterlini M. Andrei F. De Pascalis L. Palomba S. (2015). Effects of assisted reproductive technology and of women’s quality of life on depressive symptoms in the early postpartum period: a prospective case-control study. *Gynecol. Endocrinol.* 31 374–378. 10.3109/09513590.2014.1000850 25625377

[B67] Moor B. G. Macks Z. A. Güroglu B. Rombouts S. A. Molen M. W. Crone E. A. (2012). Neurodevelopmental changes of reading the mind in the eyes. *Soc. Cogn. Affect. Neurosci.* 7 44–52. 10.1093/scan/nsr020 21515640 PMC3252628

[B68] Morris A. S. Silk J. S. Steinberg L. Myers S. S. Robinson L. R. (2007). The role of the family context in the development of emotion regulation. *Soc. Dev.* 16 361–388. 10.1111/j.1467-9507.2007.00389.x 19756175 PMC2743505

[B69] Mueller I. Tronick E. (2019). Early life exposure to violence: Developmental consequences on brain and behavior. *Front. Behav. Neurosci.* 13:156. 10.3389/fnbeh.2019.00156 31338031 PMC6629780

[B70] Nolen-Hoeksema S. Wisco B. E. Lyubomirsky S. (2008). Rethinking rumination. *Perspect. Psychol. Sci.* 3 400–424. 10.1111/j.1745-6924.2008.00088.x 26158958

[B71] O’Donnell K. J. Meaney M. J. (2017). Fetal origins of mental health: The developmental origins of health and disease hypothesis. *Am. J. Psychiatry* 174 319–328. 10.1176/appi.ajp.2016.16020138 27838934

[B72] Orchard F. Reynolds S. (2018). The combined influence of cognitions in adolescent depression: Biases of interpretation, self-evaluation, and memory. *Br. J. Clin. Psychol.* 57 420–435. 10.1111/bjc.12184 29799126 PMC6175080

[B73] Parsons V. L. (2017). “Stratified Sampling,” in *Wiley statsref: Statistics reference online*, eds Balakrishnan N. Colton T. Everitt B. Piegorsch W. Ruggeri F. Teugels J. L. (Hoboken, NJ: John Wiley & Sons). 10.1002/9781118445112.stat05999.pub2

[B74] Patenaude B. Smith S. M. Kennedy D. Jenkinson M. (2011). Bayesian model of shape and appearance for subcortical brain. *NeuroImage* 56 907–922. 10.1016/j.neuroimage.2011.02.046 21352927 PMC3417233

[B75] Pendry P. Adam E. K. (2013). Child-related interparental conflict in infancy predicts child cognitive functioning in a nationally representative sample. *J. Child Family Stud.* 22 502–515. 10.1007/s10826-012-9603-3

[B76] Peverill M. Sheridan M. A. Busso D. S. McLaughlin K. A. (2019). Atypical prefrontal-amygdala circuitry following childhood exposure to abuse: Links with adolescent psychopathology. *Child Maltreat.* 24 411–423. 10.1177/1077559519852676 31146576 PMC6813859

[B77] Philip N. S. Sweet L. H. Tyrka A. R. Price L. H. Bloom R. F. Carpenter L. L. (2013). Decreased default network connectivity is associated with early life stress in medication-free healthy adults. *Eur. Neuropsychopharmacol.* 23 24–32. 10.1016/j.euroneuro.2012.10.008 23141153 PMC3581700

[B78] Power J. D. Mitra A. Laumann T. O. Snyder A. Z. Schlaggar B. L. Petersen S. E. (2014). Methods to detect, characterize, and remove motion artifact in resting state fMRI. *NeuroImage* 84 320–341. 10.1016/j.neuroimage.2013.08.048 23994314 PMC3849338

[B79] Pruim R. H. R. Mennes M. van Rooij D. Llera A. Buitelaar J. K. Christian F. (2015). ICA-AROMA: A robust ICA-based strategy for removing motion artifacts from fMRI data. *NeuroImage* 112 267–277. 10.1016/j.neuroimage.2015.02.064 25770991

[B80] R Core Team (2022). *R: A Language and Environment for Statistical Computing.* Vienna, Austria: R Foundation for Statistical Computing.

[B81] Raichle M. E. MacLeod A. M. Snyder A. Z. Powers W. J. Gusnard D. A. Shulman G. L. (2001). A default mode of brain function. *Proc. Natl. Acad. Sci. U.S.A.* 98 676–682. 10.1073/pnas.98.2.676 11209064 PMC14647

[B82] Rattaz V. Puglisi N. Tissot H. Favez N. (2022). Associations between parent–infant interactions, cortisol and vagal regulation in infants, and socioemotional outcomes: A systematic review. *Infant Behav. Dev.* 67:101687. 10.1016/j.infbeh.2022.101687 35051834

[B83] Reuben A. Moffitt T. E. Caspi A. Belsky D. W. Harrington H. Schroeder F. (2016). Lest we forget: Comparing retrospective and prospective assessments of adverse childhood experiences in the prediction of adult health. *J. Child Psychol. Psychiatry* 57 1103–1112. 10.1111/jcpp.12621 27647050 PMC5234278

[B84] Salgado S. Kaplitt M. G. (2015). The nucleus accumbens: A comprehensive review. *Stereot. Funct. Neurosurg.* 93 75–93. 10.1159/000368279 25720819

[B85] Santangelo V. Cavallina C. Colucci P. Santori A. Macri S. Mcgaugh J. L. (2018). Enhanced brain activity associated with memory access in highly superior autobiographical memory. *Proc. Natl. Acad. Sci. U.S.A.* 115 7795–7800. 10.1073/pnas.1802730115 29987025 PMC6064994

[B86] Sato J. R. Salum G. A. Gadelha A. Crossley N. Vieira G. Manfro G. G. (2015). Default mode network maturation and psychopathology in children and adolescents. *J. Child Psychol. Psychiatry* 57 55–64. 10.1111/jcpp.12444 26111611

[B87] Scheinost D. Spann M. N. McDonough L. Peterson B. S. Monk C. (2020). Associations between different dimensions of prenatal distress, neonatal hippocampal connectivity, and infant memory. *Neuropsychopharmacology* 45 1272–1279. 10.1038/s41386-020-0677-0 32305039 PMC7297970

[B88] Shih C. W. Chang C. H. (2021). Medial or lateral orbitofrontal cortex activation during fear extinction differentially regulates fear renewal. *Behav. Brain Res.* 412:113412. 10.1016/j.bbr.2021.113412 34118296

[B89] Short A. K. Baram T. Z. (2019). Early-life adversity and neurological disease: Age-old questions and novel answers. *Nat. Rev. Neurol.* 15 657–669. 10.1038/s41582-019-0246-5 31530940 PMC7261498

[B90] Sicorello M. Schmahl C. (2021). Emotion dysregulation in borderline personality disorder: A fronto–limbic imbalance? *Curr. Opin. Psychol.* 37:7. 10.1016/j.copsyc.2020.12.002 33422855

[B91] Smith K. E. Pollak S. D. (2020). Early life stress and development: Potential mechanisms for adverse outcomes. *J. Neurodevel. Dis.* 12:34. 10.1186/s11689-020-09337-y 33327939 PMC7745388

[B92] Spangler G. Zimmerman P. (2014). Emotional and adrenocortical regulation in early adolescence: Prediction by attachment security and disorganization in infancy. *Int. J. Behav. Dev.* 38 142–154. 10.1177/0165025414520808

[B93] Spanier G. B. (1976). Measuring dyadic adjustment: New scales for assessing the quality of marriage and similar dyads. *J. Marriage Family* 38 15–28. 10.2307/350547

[B94] Squire L. R. (1992). Memory and the hippocampus: A synthesis from findings with rats, monkeys, and humans. *Psychol. Rev.* 99 195–231. 10.1037/0033-295X.99.2.195 1594723

[B95] Stevens F. L. Hurley R. A. Taber K. H. (2011). Anterior cingulate cortex: Unique role in cognition and emotion. *J. Neuropsychiatry Clin. Neurosci.* 23 121–125. 10.1176/jnp.23.2.jnp121 21677237

[B96] Vänskä M. Punamäki R.-L. Tolvanen A. Lindblom J. Flykt M. Unkila-Kallio L. (2011). Maternal pre-and postnatal mental health trajectories and child mental health and development: Prospective study in a normative and formerly infertile sample. *Int. J. Behav. Dev.* 35 517–531. 10.1177/0165025411417505

[B97] VanTieghem M. R. Tottenham N. (2018). Neurobiological programming of early life stress: Functional development of amygdala-prefrontal circuitry and vulnerability for stress-related psychopathology. *Curr. Top. Behav. Neurosci.* 38 117–136. 10.1007/7854_2016_42 28439771 PMC5940575

[B98] Weeks M. Coplan R. J. Ooi L. L. (2017). Cognitive biases among early adolescents with elevated symptoms of anxiety, depression, and co-occurring symptoms of anxiety-depression: Cognitive biases, anxiety, and depression. *Infant Child Dev.* 26:2011. 10.1002/icd.2011

[B99] Whitfield-Gabrieli S. Ford J. M. (2012). Default mode network activity and connectivity in psychopathology. *Ann. Rev. Clin. Psychol.* 8 49–76. 10.1146/annurev-clinpsy-032511-143049 22224834

[B100] Wikman P. Moisala M. Ylinen A. Lindblom J. Leikas S. Salmela-Aro K. (2021). Brain responses to peer feedback in social media are modulated by valence and personality dimensions in late adolescence. *Front. Behav. Neurosci.* 16:790478. 10.3389/fnbeh.2022.790478 35706832 PMC9190756

[B101] Wu Y. Lu Y. C. Jacobs M. Pradhan S. Kapse K. Zhao L. (2020). Association of prenatal maternal psychological distress with fetal brain growth, metabolism, and cortical maturation. *JAMA Network Open* 3:e1919940. 10.1001/jamanetworkopen.2019.19940 31995213 PMC6991285

[B102] Xu X. Yuan H. Lei X. (2016). Activation and connectivity within the default mode network contribute independently to future-oriented thought. *Sci. Rep.* 6:21001. 10.1038/srep21001 26867499 PMC4751480

[B103] Yan C. G. Chen X. Li L. Castellanos F. X. Bai T. J. Bo Q. J. (2019). Reduced default mode network functional connectivity in patients with recurrent major depressive disorder. *Proc. Natl. Acad. Sci. U.S.A.* 116 9078–9083. 10.1073/pnas.1900390116 30979801 PMC6500168

[B104] Yeshurun Y. Nguyen M. Hasson U. (2021). The default mode network: Where the idiosyncratic self meets the shared social world. *Nat. Rev. Neurosci.* 22 181–192. 10.1038/s41583-020-00420-w 33483717 PMC7959111

[B105] Zeev-Wolf M. Levy J. Goldstein A. Zagoory-Sharon O. Feldman R. (2019). Chronic early stress impairs default mode network connectivity in preadolescents and their mothers. *Biol. Psychiatry Cogn. Neurosci. Neur.* 4 72–80. 10.1016/j.bpsc.2018.09.009 30446436

[B106] Zhou H.-X. Chen X. Shen Y.-Q. Li L. Chen N.-X. Zhu Z.-C. (2020). Rumination and the default mode network: Meta-analysis of brain imaging studies and implications for depression. *NeuroImage* 206:116287. 10.1016/j.neuroimage.2019.116287 31655111

[B107] Zhou N. Cao H. Leerkes E. M. (2017). Interparental conflict and infants’ behavior problems: The mediating role of maternal sensitivity. *J. Family Psychol.* 31 464–474. 10.1037/fam0000288 28114771 PMC5449209

[B108] Zhu Y. Gao H. Tong L. Li Z. Wang L. Zhang C. (2019). Emotion regulation of hippocampus using real-time fmri neurofeedback in healthy human. *Front. Hum. Neurosci.* 13:242. 10.3389/fnhum.2019.00242 31379539 PMC6660260

