# Supplementary material for: Early life stress is associated with the default mode and fronto-limbic network connectivity among young adults
Source: Front Behav Neurosci. 2022 Sep 23;16:958580. doi: 10.3389/fnbeh.2022.958580 (PMC9537946; doi:10.3389/fnbeh.2022.958580)

# Supplementary Material


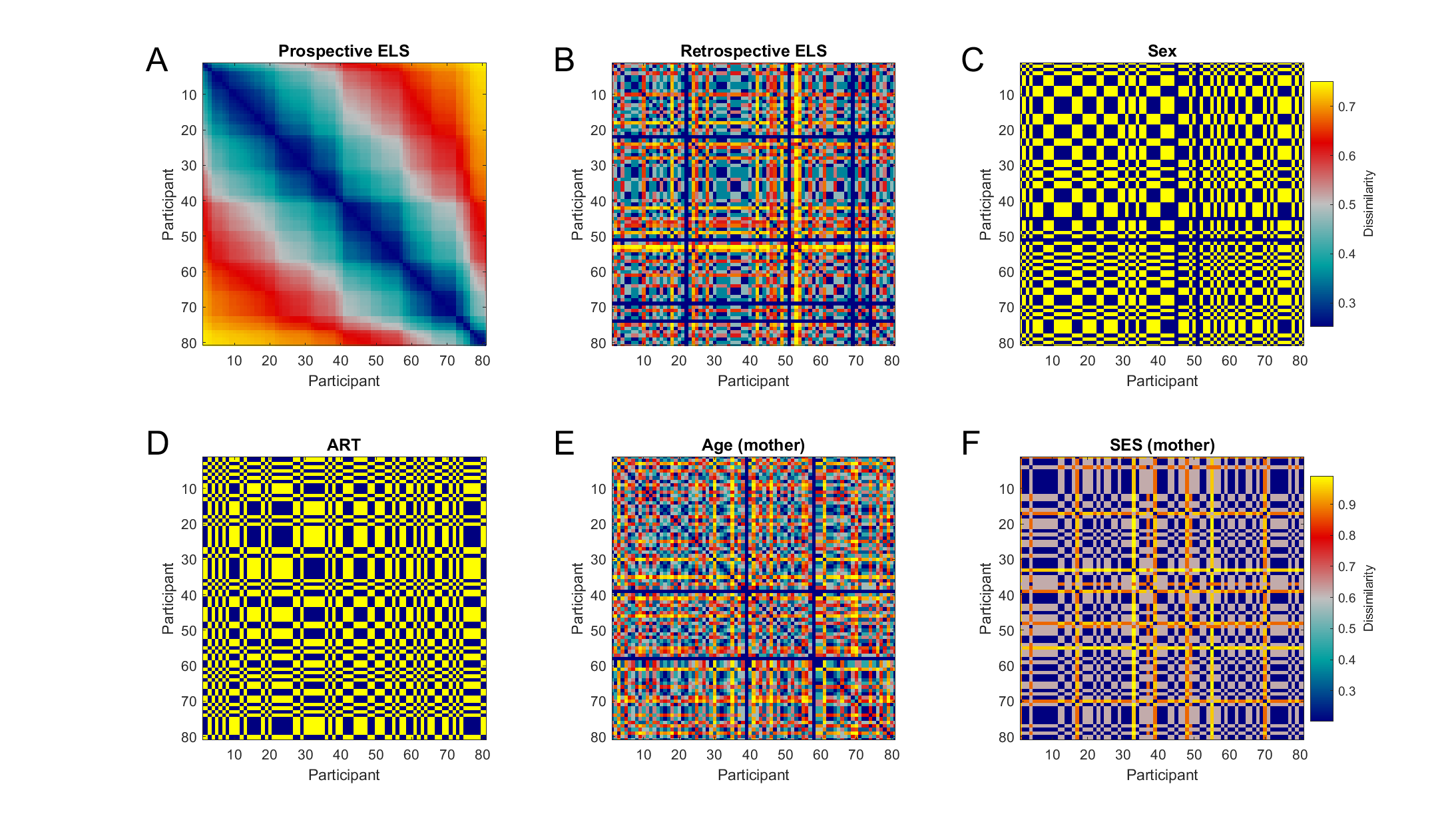


**Supplementary Figure 1.** Representational dissimilarity matrices for independent variables representing dissimilarities between all pairwise comparisons across all participants (81×81). Colors indicate the extent of dissimilarity between participants, where dark blue represents maximum similarity and yellow represents maximum dissimilarity. Representational dissimilarity matrices are displayed for (A) prospective ELS, (B) retrospective ELS, (C) participant sex, (D) assisted reproduction therapy (ART), (E) mother’s age, and (F) mother’s socio-economic status (SES). Note that matrices are sorted according to prospective ELS.


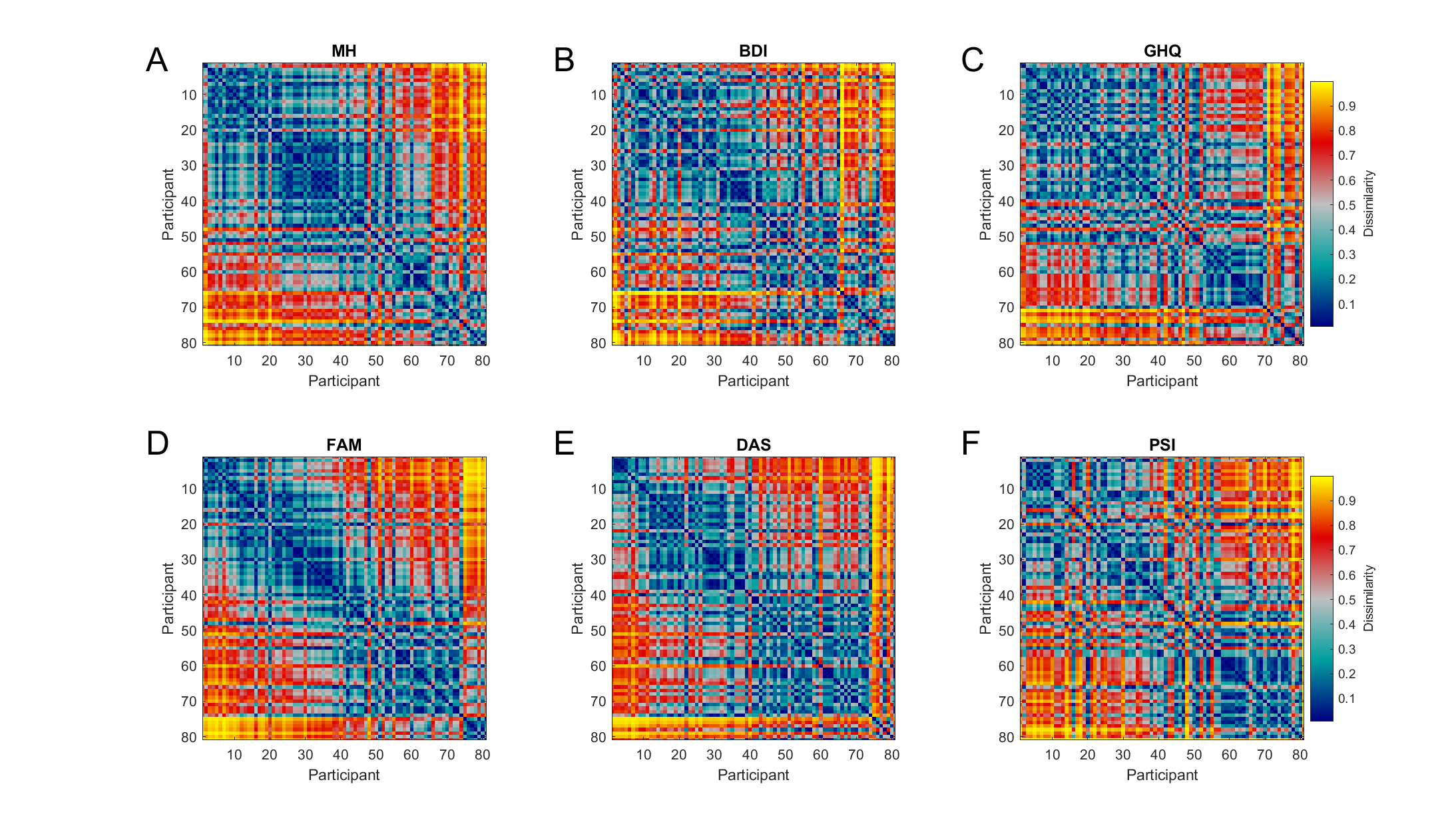


**Supplementary Figure 2.** Representational dissimilarity matrices for prospective ELS components representing dissimilarities between all pairwise comparisons across all participants (81×81). Colors indicate the extent of dissimilarity between participants, where dark blue represents maximum similarity and yellow represents maximum dissimilarity. Representational dissimilarity matrices are displayed for (A) prospective mental health problem domain (MH) and (D) family-related problem domain (FAM)s, as well as for individual questionnaires: (B) Beck’s Depression Inventory (BDI), (C) General Health Questionnaire (GHQ), (E), Dyadic Adjustment Scale (DAS), and (F) Parenting Stress Index (PSI). Note that the MH variable comprises BDI and GHQ scores, and the FAM variable comprises DAS and PSI scores. Note that matrices are sorted according to the prospective ELS variable (see supplement Figure 1A).


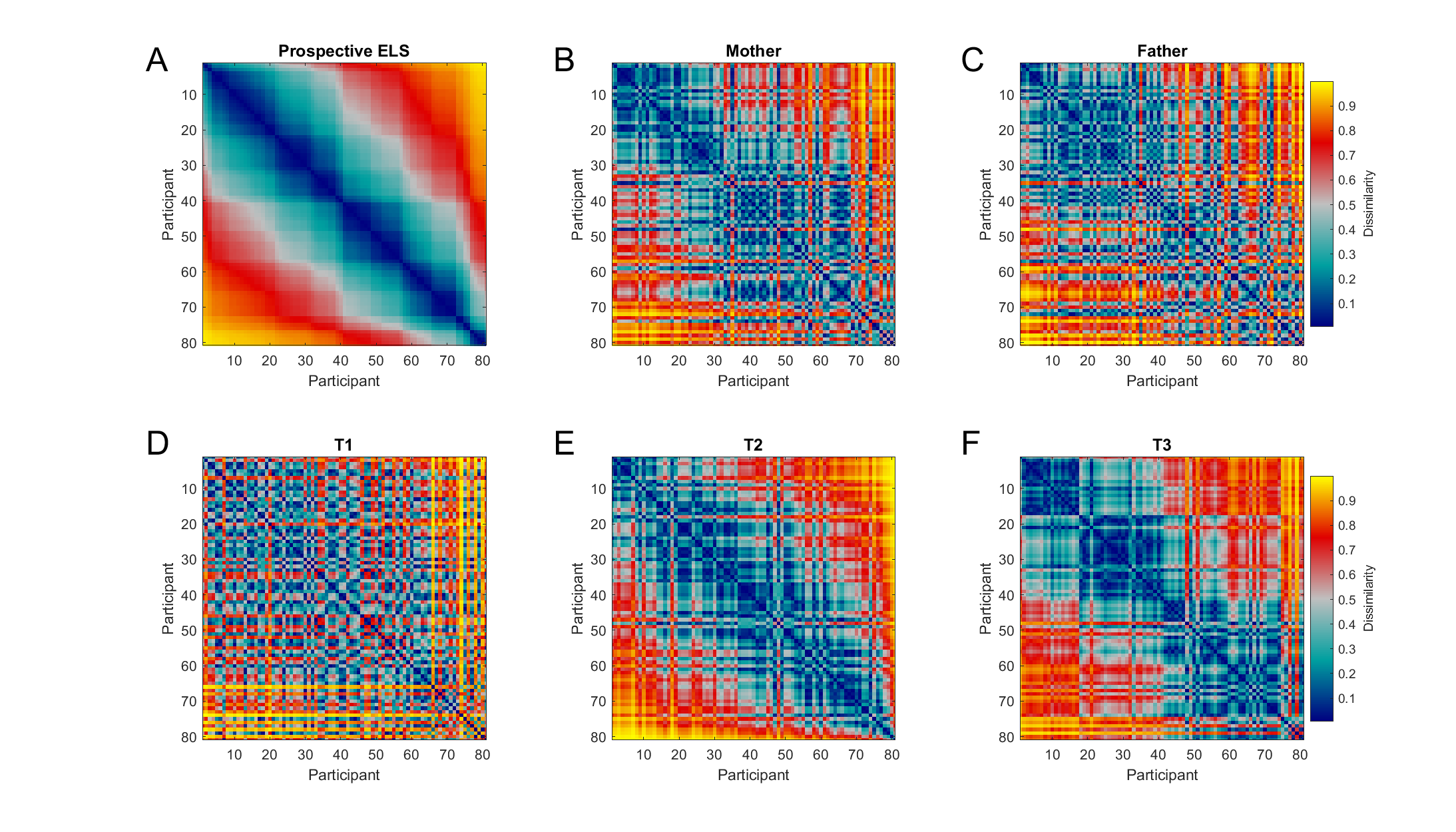


**Supplementary Figure 3.** Representational dissimilarity matrices for prospective ELS components representing dissimilarities between all pairwise comparisons across all participants (81×81). Colors indicate the extent of dissimilarity between participants, where dark blue represents maximum similarity and yellow represents maximum dissimilarity. Representational dissimilarity matrices are displayed for the total prospective ELS score (A), prospective ELS scores for both the mother (B) and the father (C), and prospective ELS scores from the three time points T1 (pregnancy; D), T2 (two months after birth; E), and T3 (12 months after birth; F). Note that matrices are sorted according to the prospective ELS variable.

**Supplementary Figure 4.** Partial correlation of DMN (A) and FLN (B) network profile dissimilarities with dissimilarity matrices of mental health (MH) and family (FAM) related problems, with participant sex, assisted reproduction therapy group (ART), mother’s age, and mother’s socio-economic status (SES) partialled out. Colors indicate the strength and direction of correlation between the dissimilarity matrices of variables, where blue represents negative correlations and red and yellow represents positive correlations. *p<.05, **p<.01, ***p<.001.


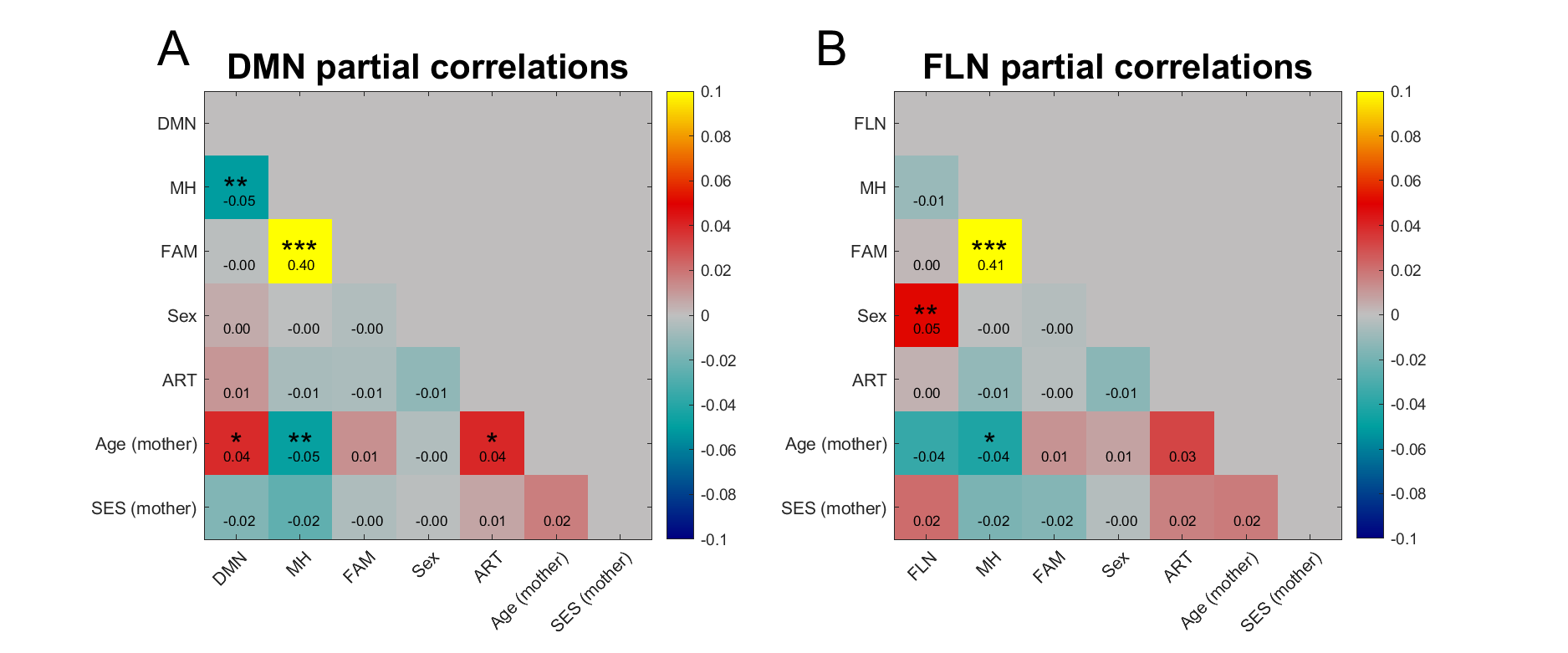


**Supplementary Figure 5.** Partial correlation of DMN (A) and FLN (B) network profile dissimilarities with dissimilarity matrices of individual questionnaires BDI, GHQ, DAS, and PSI, with participant sex, assisted reproduction therapy group (ART), mother’s age, and mother’s socio-economic status (SES) partialled out. Colors indicate the strength and direction of correlation between the dissimilarity matrices of the variables, where blue represents negative correlations and red and yellow represents positive correlations. *p<.05, **p<.01, ***p<.001.


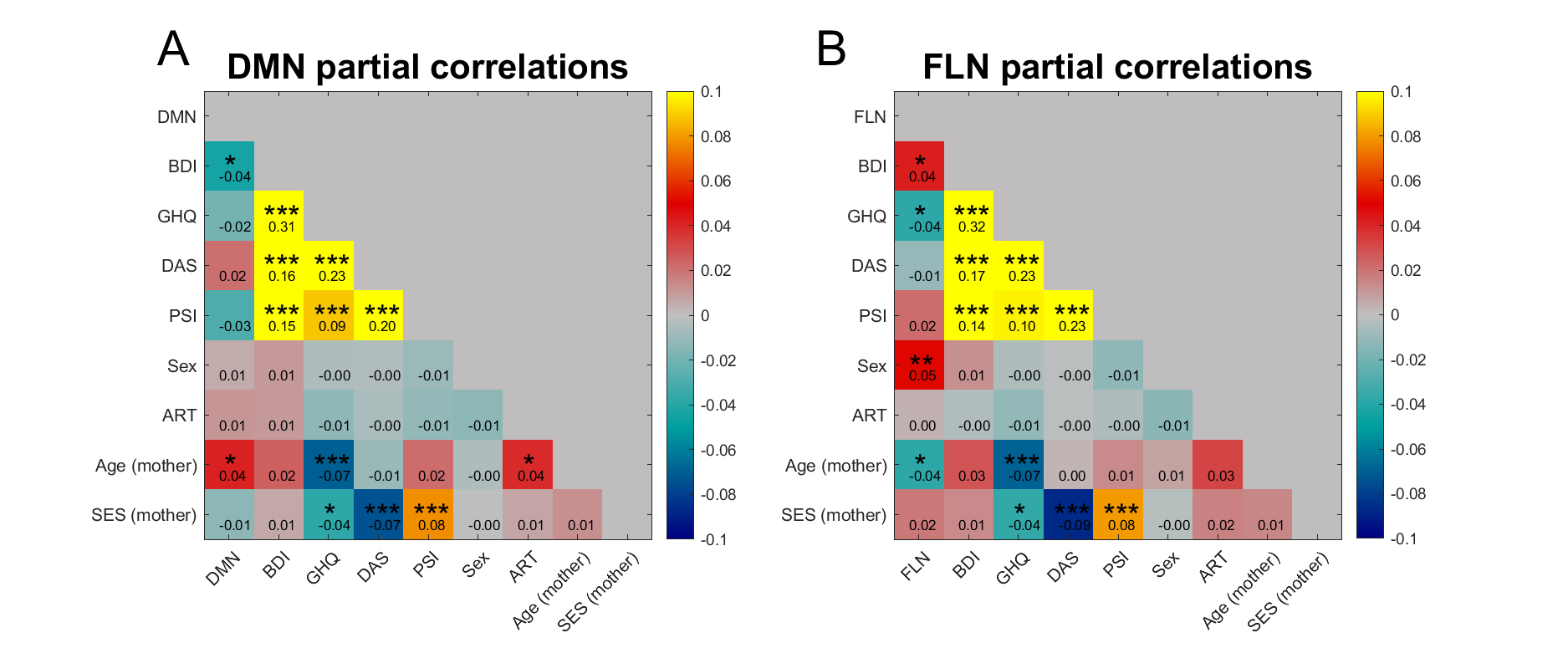


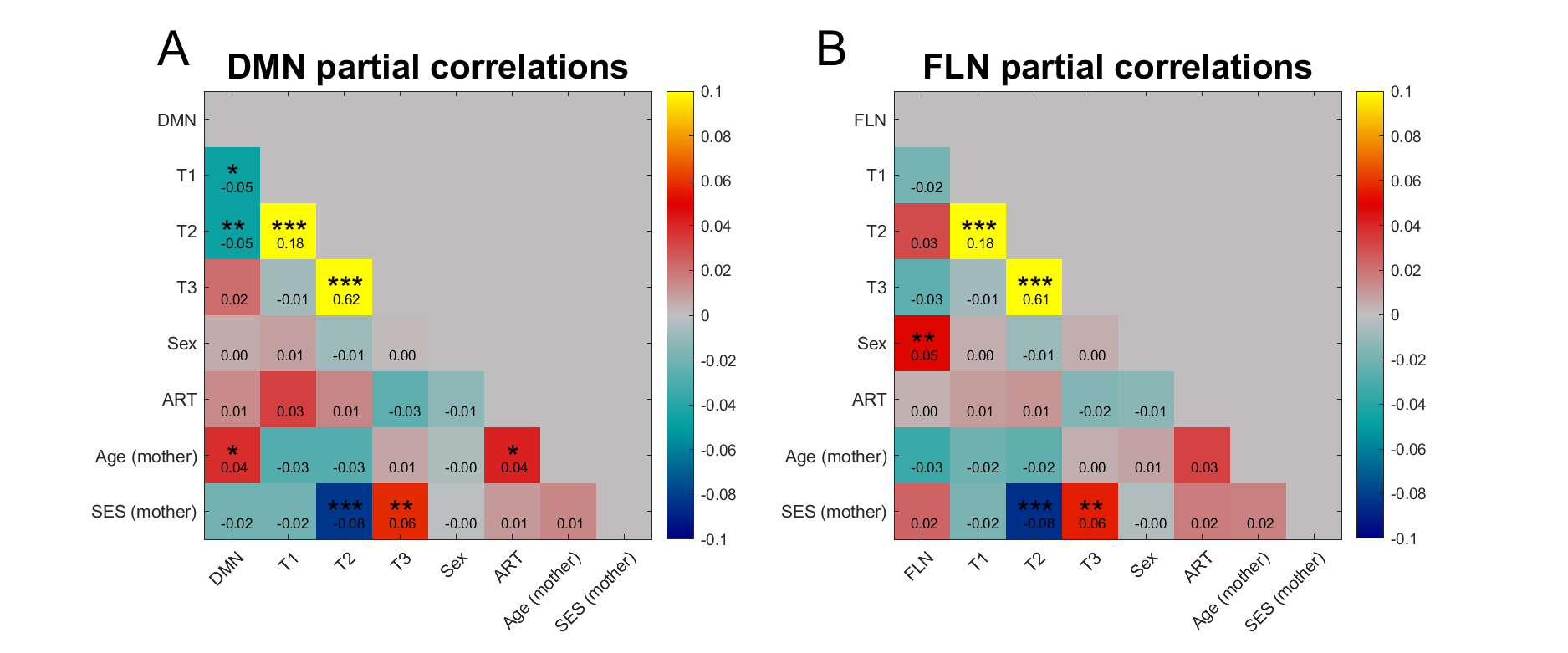
**Supplementary Figure 6.** Partial correlation of DMN (A) and FLN (B) network profile dissimilarities with dissimilarity matrices of T1, T2, and T3, with participant sex, assisted reproduction therapy group (ART), mother’s age, and mother’s socio-economic status (SES) partialled out. Colors indicate the strength and direction of correlation between the dissimilarity matrices of the variables, where blue represents negative correlations and red and yellow represents positive correlations. *p<.05, **p<.01, ***p<.001.


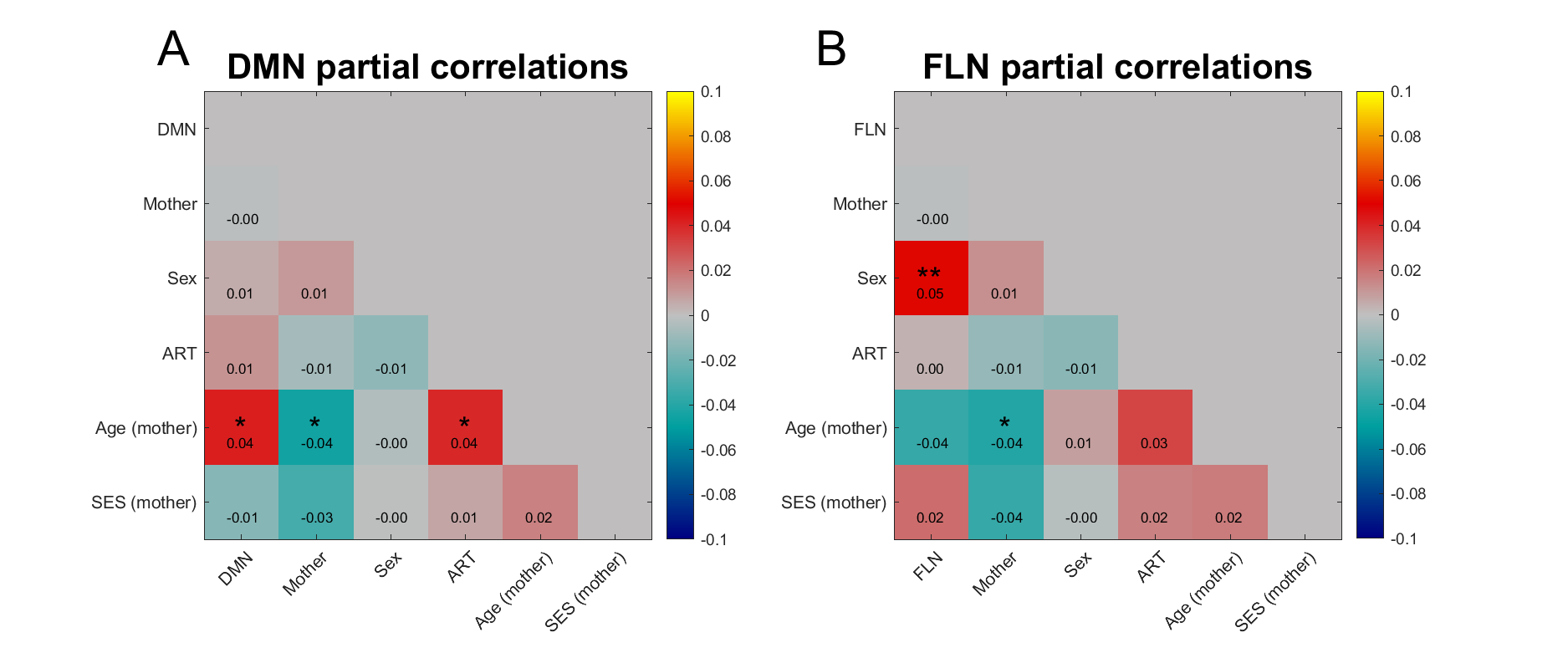


**Supplementary Figure 7.** Partial correlation of DMN (A) and FLN (B) network profile dissimilarities with the prospective ELS scores of the mother only, with participant sex, assisted reproduction therapy group (ART), mother’s age, and mother’s socio-economic status (SES) partialled out. Colors indicate the strength and direction of correlation between the dissimilarity matrices of the variables, where blue represents negative correlations and red and yellow represents positive correlations. *p<.05, **p<.01.

**Supplementary Figure 8.** Partial correlation of DMN (A) and FLN (B) network profile dissimilarities with the prospective ELS scores of the father only, with participant sex, assisted reproduction therapy group (ART), mother’s age, and mother’s socio-economic status (SES) partialed out. Colors indicate the strength and direction of correlation between the dissimilarity matrices of the variables, where blue represents negative correlations and red and yellow represents positive correlations. *p<.05, **p<.01.


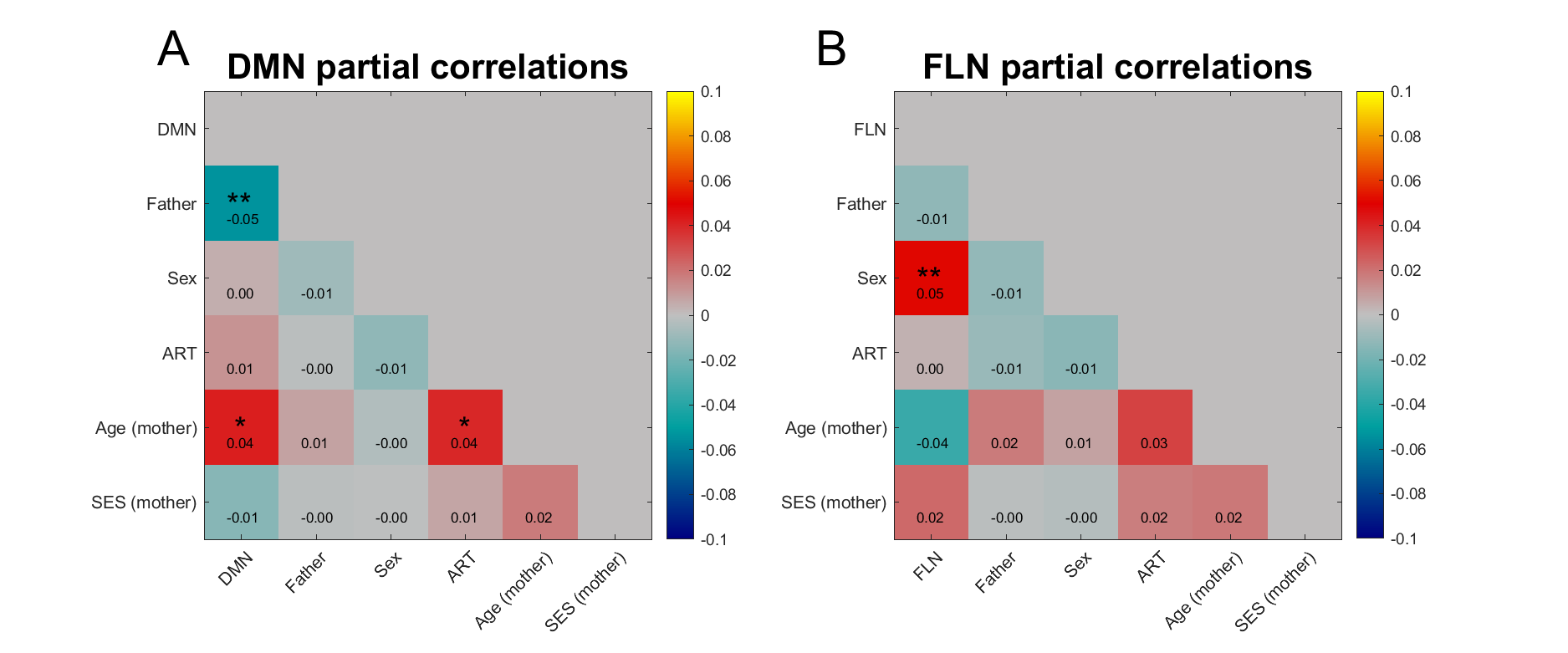

Supplement: Supplementary file 1 [file Data_Sheet_1.docx]
